# Membrane vesicles in *Acidithiobacillia* class extreme acidophiles: influence on collective behaviors of ‘*Fervidacidithiobacillus caldus*’

**DOI:** 10.3389/fmicb.2023.1331363

**Published:** 2024-01-26

**Authors:** Stefano Rossoni, Simón Beard, María Ignacia Segura-Bidermann, Juan Duarte-Ramírez, Francisco Kirhman Osorio, Manuel Varas-Godoy, Patricio Martínez-Bellange, Mario Vera, Raquel Quatrini, Matías Castro

**Affiliations:** ^1^Centro Científico y Tecnológico Ciencia & Vida, Fundación Ciencia & Vida, Santiago, Chile; ^2^Facultad de Medicina y Ciencia, Universidad San Sebastián, Santiago, Chile; ^3^Departamento de Oceanografía, Facultad de Ciencias Naturales y Oceanográficas, Universidad de Concepción, Concepción, Chile,; ^4^Instituto de Ingeniería Biológica y Médica, Escuelas de Ingeniería, Medicina y Ciencias Biológicas, Pontificia Universidad Católica de Chile, Santiago, Chile; ^5^Cancer Cell Biology Lab., Centro de Biología Celular y Biomedicina (CEBICEM), Facultad de Medicina y Ciencia, Universidad San Sebastián, Santiago, Chile; ^6^Biomining Advisor, Santiago, Chile; ^7^Departamento de Ingeniería de Minería, Escuela de Ingeniería, Pontificia Universidad Católica de Chile, Santiago, Chile; ^8^Instituto Milenio de Oceanografía (IMO), Universidad de Concepción, Concepción, Chile

**Keywords:** outer membrane vesicles (OMVs), *Acidithiobacillus*, surface colonization, biofilm, swarming, adhesins, sulfur oxidation, tube-shaped membranous structures (TSMS)

## Abstract

Membrane vesicles (MVs) are envelope-derived extracellular sacs that perform a broad diversity of physiological functions in bacteria. While considerably studied in pathogenic microorganisms, the roles, relevance, and biotechnological potential of MVs from environmental bacteria are less well established. *Acidithiobacillaceae* family bacteria are active players in the sulfur and iron biogeochemical cycles in extremely acidic environments and drivers of the leaching of mineral ores contributing to acid rock/mine drainage (ARD/AMD) and industrial bioleaching. One key aspect of such a role is the ability of these bacteria to tightly interact with the mineral surfaces and extract electrons and nutrients to support their chemolithotrophic metabolism. Despite recent advances in the characterization of acidithiobacilli biofilms and extracellular matrix (ECM) components, our understanding of its architectural and mechanistic aspects remains scant. Using different microscopy techniques and nano-tracking analysis we show that vesiculation is a common phenomenon in distant members of the *Acidithiobacillaceae* family, and further explore the role of MVs in multicellular colonization behaviors using ‘*Fervidacidithiobacillus caldus*’ as a bacterial model. Production of MVs in ‘*F. caldus*’ occurred in both planktonic cultures and biofilms formed on sulfur surfaces, where MVs appeared individually or in chains resembling tube-shaped membranous structures (TSMSs) important for microbial communication. Liquid chromatography–mass spectrometry data and bioinformatic analysis of the MV-associated proteome revealed that ‘*F. caldus*’ MVs were enriched in proteins involved in cell–cell and cell–surface processes and largely typified the MVs as outer MVs (OMVs). Finally, microbiological assays showed that amendment of ‘*F. caldus*’ MVs to cells and/or biofilms affects collective colonizing behaviors relevant to the ecophysiology and applications of these acidophiles, providing grounds for their exploitation in biomining.

## 1 Introduction

The bacterial family *Acidithiobacillaceae* is composed of extremely acidophilic chemolithoautotrophic bacteria, growing optimally at pH values below 3 (Moya-Beltrán et al., 2021). Members of this family obtain energy from the oxidation of reduced inorganic sulfur compounds (RISCs), while some of them can also catalyze the oxidation of ferrous iron or hydrogen. The main products of this chemolithotrophic metabolism are (sulfuric) acid (H^+^) and ferric iron, which are the principal leaching agents of metal sulfides. Microbial oxidation of metal sulfides has been used for decades by the mining industry as a biotechnological process for metal extraction known as biomining, allowing the recovery of economically relevant elements from low-grade ores (Johnson, 2014). On the other hand, oxidation of metal sulfides promotes the formation of highly contaminating metal-loaded acidic waters that drain from mines (acid mine drainage, AMD) or air-exposed ores (acid rock drainage, ARD) and constitute one of the most serious threats to water resources, being labeled by the United Nations as the second biggest environmental problem after global warming (Tuffnell, 2017).

Besides mining sites (mine heaps, waste rock dumps, abandoned mines, acidic pit lakes, etc.), bacteria belonging to *Acidithiobacillaceae* family colonize diverse natural environments including geothermal sites, acidic sulfur-springs, acidic rivers in volcanic areas, sulfidic caves, acid sulfate soils, and coastal acid sulfate soils as well as sulfide-rich rock strata in Antarctica (Hedrich and Schippers, 2021). These habitats are shaped by the (bio)chemical reactions occurring at mineral–water interface (Vriens et al., 2020), many of which are microbiologically driven. Two different cell populations are relevant in this context: the planktonic and the substrate-attached cells. Generally, the planktonic state involves individual cells, whereas surface-associated cells could develop multicellular structures through swarming motility and biofilm development (Kearns, 2010; Sauer et al., 2022). The transition of these two main lifestyles involves complex molecular mechanisms of signal transduction and cell–cell communication, many of which have been studied in this group of acidophiles (Bellenberg et al., 2014; Moya-Beltrán et al., 2019; Castro et al., 2020; Díaz et al., 2021).

Swarming motility, the collective locomotion powered by the rotation of the flagella, has been poorly studied in *Acidithiobacillaceae* family members. Apart from the bioinformatic identification and/or characterization of the flagellar gene clusters in a couple of species of the family, few efforts have been made to understand swarming behavior under acidic conditions (Castro et al., 2015; Yang et al., 2019). In turn, biofilm formation has been studied in further depth in some *Acidithiobacillaceae*. As in other bacteria, this has been shown to be a dynamic process that requires a self-produced extracellular matrix (ECM). The dependence of cell attachment and biofilm development on ECM has been demonstrated for the mesophilic iron-oxidizer *Acidithiobacillus ferrooxidans* (Sand and Gehrke, 2006; Vera et al., 2013), the mesophilic sulfur-oxidizer *Acidithiobacillus thiooxidans* (Harneit et al., 2006; González et al., 2012), and the moderate thermophilic sulfur-oxidizer ‘*Ferviacidithiobacillus caldus*’ (ex. *Acidithiobacillus caldus*) (Edwards et al., 2000; Castro et al., 2020; Moya-Beltrán et al., 2021). The ECM in these bacteria is commonly composed of a mixture of fatty acids, proteins, and sugars, with variations depending on the type of substrate (Gehrke et al., 1998) and the taxon (Castro et al., 2015, 2020). The biofilm structure has also been observed to be dependent on substrate. In contrast to metal sulfides, where the cells tend to form monolayer biofilms (González et al., 2012; Bellenberg et al., 2014), biofilms formed by ‘*F. caldus*’ and *A. thiooxidans* on elemental sulfur develop structures of further complexity consisting in a few layers of cells connected through filament-like projections of yet-unknown nature (Castro et al., 2015; Díaz et al., 2018). Despite these advances, our knowledge about the ECM produced by the *Acidithiobacilliaceae* is still very limited, particularly regarding the structural organization of its basic components, as well as the physiological and molecular mechanisms in which the matrix is involved.

Membrane vesicles (MVs) are nanosized proteoliposomes derived from the cell membrane. Their shedding from the cell surface or ‘vesiculation’ is a conserved phenomenon occurring across all domains of life (Deatherage and Cooksona, 2012; Bonnington and Kuehn, 2014; Haurat et al., 2015). In recent years, representatives of the main branches of the tree of life have been demonstrated to produce MVs (Gill et al., 2019; Liu et al., 2021a). MVs participate in diverse and critical functions in microbial physiology, such as host colonization and pathogenesis (Kuehn and Kesty, 2005), adaptive responses to stress (MacDonald and Kuehn, 2012), resistance to bacteriophage infection (Manning and Kuehn, 2011), antibiotics and antibodies sequestering (Chattopadhyay and Jaganandham, 2015), bacterial mortality (Kadurugamuwa and Beveridge, 1996), gene transfer (Domingues and Nielsen, 2017), and nutrient cycling (Liu et al., 2021b). Importantly, in a few bacterial species it has been demonstrated that MVs play structural and regulatory roles in biofilm formation (Baumgarten et al., 2012), cell–cell association (Schooling and Beveridge, 2006; Remis et al., 2014; Wang et al., 2015), and interaction with ECM components such as exopolysaccharides and extracellular DNA (eDNA) (Schooling et al., 2009).

Previous reports have provided hints on the production of MVs in *Acidithiobacilliaceae* family members. Knickerbocker described the release of ‘blebs’ from a sulfur-oxidizing bacteria, phenotypically identified as *A. thiooxidans* (Knickerbocker et al., 2000). No formal characterization of the presumed MVs was presented in that study, yet it was conjectured that blebs could have a role in ‘wetting’ elemental sulfur, which is highly hydrophobic (Jones and Starkey, 1961), thus facilitating bacterial colonization and oxidation of sulfur particles. More recently, Covarruvias et al. described that under DNA-damaging conditions, a culture of ‘*F. caldus*’ strain ATCC 51756, which carries a Myoviridae prophage integrated in its genome, produced DNA-containing tail-less icosahedral viral capsids and spherical outer membrane vesicles (Covarrubias et al., 2017). While the isolated viruses were non-infective (Covarrubias et al., 2018), the outer membrane vesicles present in the viral filtrates coincided in size and shape with those described for MVs. Despite these findings, the production of MVs by these and other members of the *Acidithiobacillaceae* family has not been confirmed, and the ecophysiological roles of MVs in this group of extreme acidophiles are still unknown. In this study, we evaluate the production of MVs in *Acidithiobacillaceae* family members and explore their role in multicellular colonization behaviors, such as swarming motility and biofilm development.

## 2 Materials and methods

### 2.1 Bacteria, cultivation, and incubation conditions

‘*F. caldus*’ ATCC 51756^T^, *A. thiooxidans* ATCC 19377^T^, and *A. ferrooxidans* ATCC 23270^T^ liquid cultures were grown in modified Bangor Acidophile Research Team (BART) medium plus trace elements solution (Ňancucheo et al., 2016). The modified medium (1X) lacked yeast extract, glycerol, and zinc, and the pH was adjusted to 2.5 or 2.8 when supplemented with elemental sulfur (1% w/v) or potassium tetrathionate (K_2_S_4_O_6_) (0.15% w/v) as solid and soluble energy sources, respectively. Flasks for cell biomass production and bulk production of MVs were incubated on rotary shakers at 100 rpm and 30°C, except ‘*F. caldus*’ ATCC 51756^T^ cultures which were grown at 40°C. Growth was monitored periodically by cell counting using an Improved Neubauer chamber (0.02 mm depth, Marienfeld GmbH & Co, Germany) and an optical microscope with phase contrast (Zeiss Axioskop 2). Bulk production of MVs by the three strains for initial observation, size determination, and quantification (section 3.1) were assessed in sulfur-grown 10-day-old cultures (~4 × 10^8^ cells/mL) grown under the above-stated conditions. To test the effect of temperature on production of MVs, ‘*F. caldus*’ sulfur-grown stationary phase cultures were resuspended in 600 mL (~2.6 × 10^9^ cells/mL) and disposed in three flasks. Each flask (200 mL, ~5 × 10^11^ cells/mL) was incubated at physiological temperature (40°C) or non-optimal temperatures (55°C and 70°C) for 8 h at 100 rpm, in duplicate before MVs harvesting and analysis. ‘*F. caldus*’ ATCC 51756^T^ cells used in swarming experiments were harvested from tetrathionate-grown cultures at mid-logarithmic (~8 × 10^7^ cells/mL) or late logarithmic/early stationary phase (~1.3 × 10^8^ cells/mL). To conduct the swarming motility tests, a phytagel–tetrathionate semisolid medium (PTSM) was developed, containing phytagel (Sigma) (0.07% w/v) as gelling agent and tetrathionate (0.15% w/v) as energy source (see Supplementary Methods). Biofilm formation on sulfur was conducted as described in Castro et al. (2015) by incubating homemade sulfur coupons with ‘*F. caldus*’ ATCC 51756^T^ cells for 72 h at 40°C. Coupons were washed twice with fresh BART medium and fixed in paraformaldehyde (4%) until observation. Biofilms were developed on a semisolid medium by adding 20 μL containing 1 × 10^8^ ‘*F. caldus*’ cells on PTSM plates containing phytagel (0.9% w/v) and tetrathionate (0.15% w/v), which were incubated at 40°C for 10 days. Biofilms were fixed in an epoxy resin for thin sectioning.

### 2.2 Membrane vesicle harvesting, isolation, and quantification

Cell cultures that reached the desired growth stage for harvest were centrifuged at 6,500 g using an F15-6 X 100Y rotor (Thermo Heraeus Megafuge 16R Centrifuge) for 15 min at 4°C to produce cell-free culture supernatants. Remnant cells were separated from the solution via vacuum filtration through 0.45- and 0.22-μm pore size filters (Millipore™, 47 mm). Next, the supernatants were concentrated to a final volume of ~60 mL through tangential flow filtration (Masterflex™ #HV-07518-00) using a 100-KDa cassette (Minimate™ TFF Capsule Omega 100 K membrane). Finally, MVs were sedimented from the concentrated supernatants through high-speed centrifugation at 150,000 g during 1.5 h at 4°C using an SW 41 rotor (Beckman Coulter) and an ultracentrifuge (Optima™ LE-80 K, Beckman Coulter). The pellets obtained were resuspended in 1 mL of filter (0.22 μm) sterilized PBS buffer (pH 5). Samples were stored at −20 or 4°C until further experiments. This protocol is a modification of those described elsewhere (Chutkan et al., 2013; Klimentová and Stulík, 2015; Busatto et al., 2018; Gerritzen et al., 2019). Fresh MV preparations and serial dilutions were used to evaluate the size distribution of MVs and for quantification using nanoparticle tracking analysis on a NanoSight NS300 (Malvern Panalytical NanoSight) equipment available at the Unit of Electron Microscopy (Universidad de Chile). The parameters (*camera level, slider shutter, slider gain, detect threshold*) were adjusted manually for data recompilation.

### 2.3 Evaluation of multicellular surface colonization behaviors

Swarming motility was tested for ‘*F. caldus*’ ATCC 51756^T^ and *A. thiooxidans* ATCC 19377^T^, yet not in *A. ferrooxidans* ATCC 23270^T^ as this strain lacks flagella-encoding genes and thus also the capacity to swarm (e.g., Valdés et al., 2008). Cells were harvested from tetrathionate-grown cultures, washed (twice), and resuspended in BART medium (pH 2.8). The effect of amendment of cognate MVs on swarming of ‘*F. caldus*’ cells was tested under two different conditions: (a) on mid-logarithmic cells (~8 × 10^7^ cells/mL) or (b) on stationary phase cells (~1.3 × 10^8^ cells/mL). Cells were resuspended at a concentration of 8 × 10^9^ cells/mL in BART medium and then mixed 1:1 with a MV-enriched cell-free extract of 1 × 10^12^ particles/mL or PBS buffer as negative control. 5 μL of samples containing cells (2 × 10^7^) or cells (2 × 10^7^) plus MVs (2.5 × 10^9^ MVs) were inoculated in the center of PTSM swarming plates. Swarming was monitored daily using a stereoscope (LEICA EZ4 W) (10 X), and photographs were taken with a camera (Canon Power Shot G12) at a focal length of 21 cm. Attachment of ‘*F. caldus*’ cells to sulfur surfaces was tested using both elemental sulfur powder (Sigma-Aldrich) and lentils (Elemental^®^). The incubation time required for maximum early attachment on sulfur surfaces was determined empirically at 120 min (see Supplementary Methods). Sulfur was incubated with or without MVs at two concentrations (1X = 7.5 × 10^9^ MVs and 10X = 7.5 × 10^10^ MVs) for 15 min (sulfur powder) or 120 min (sulfur lentils) and then exposed to mid-logarithmic/planktonic cell suspension for 120 min. The decrease in concentration of planktonic cells due to surface attachment was monitored over 3 h to avoid cell growth effects (generation time: 8 h).

### 2.4 Staining and microscopy analyses

Biofilms of ‘*F. caldus*’ ATCC 51756^T^ on sulfur coupons were observed by confocal laser scanning microscopy (CLSM) and scanning electron microscopy (SEM). For CLSM, sulfur coupons were stained with the LIVE *Bac*Light ‘live/dead’ stain according to the supplier recommendations (Thermo Fisher). A laser scanning module (LSM 510 Carl Zeiss^®^ Jena), coupled to an inverted Axiovert 100 MBP microscope (Zeiss^®^), was used. Micrographs were obtained with the plan-apochromatic 100×/0.79 oil DIC objective. Basic visualization and image manipulation were performed using ImageJ.^1^ For SEM, sulfur coupons were processed to critical point drying, coated with gold, and observed in a LEO VP1400 microscope (Faculty of Physics, Pontificia Universidad Católica de Chile).

Thin sections of colony biofilms (~80 nm) were obtained using a microtome (Advanced Microscopy Unit, Pontificia Universidad Católica de Chile), stained with uranyl acetate and examined by using Philips Tecnai 12 (Biotwin) or Talos F200C G2 Transmission Electron Microscope (TEM). Early attachment of ‘*F. caldus*’ ATCC 51756^T^ cells on sulfur lentils and the effect of amended MVs were monitored by epifluorescence microscope (EFM). MVs and cells of ‘*F. caldus*’ were differentially stained with DiO (11.3 nM) and 4′,6-diamidino-2-phenylindole (DAPI) (300 nM), respectively (see Supplementary Methods). Images were acquired with an inverted EFM microscope Zeiss^®^ Axio Observer Z1/7 using the 40 X objective and processed with the software Zeiss^®^ Zen Pro version 3.0. For massive image analysis, at least 12 tiles were taken per sample, each composed of 16 individual images. Images were taken in Z-stacking mode with a focus depth of approximately 30 layers of 0.57 μm each. EFM images were manually pre-filtered and then processed using a Python-based code, which allowed for the extraction of quantitative parameters of interest (see Supplementary Methods). The data were analyzed using GraphPad Prism software, employing standard error of mean to evaluate the differences between experimental conditions and establish correlations between variables (presence of vesicles, vesicle density, and presence of cells). The detection of nucleic acid associated with ‘*F. caldus*’ MVs was performed by CLSM. Cells (5 × 10^7^ cells/mL) and MVs (7.5 × 10^10^ particles/mL) were co-stained with DAPI (800 nM) and FM 4–64 (5 μg/mL). Samples were disposed on glass slides combined with FluorSave (FluorSave^TM^ Reagent, Merck Millipore) and visualized using a Zeiss Airyscan LSM 880 microscope with Airyscan detection.

### 2.5 Protein extraction and proteomic analysis of MVs

Protein purification and sequencing work were carried out at the Melisa Institute (Concepción, Chile). Proteins were recovered from MV-enriched cell-free extracts from two independent replicates of ‘*F. caldus*’ ATCC 51756^T^ sulfur-grown cultures (MV1 and MV2) and their integrity assessed by SDS-PAGE. Purified proteins were trypsin-digested (Promega, #V5071) and subjected to LC–MS/MS analysis using a nanoUHPLC nanoElute (Bruker Daltonics) coupled to a timsTOF Pro mass spectrometer (‘Trapped Ion Mobility Spectrometry—Quadrupole Time Of Flight Mass Spectrometer,’ Bruker Daltonics) (see Supplementary Methods). Data obtained were analyzed with the PEAKS Studio X + software (Bioinformatics Solutions). Identification of the ‘*F. caldus*’ ATCC 51756^T^ proteome was done against the UniProt database (proteome ID UP000005522, 2,867 entries). False discovery rate (FDR) estimation was included through a decoy database, with an FDR ≥ 1% (significance ≥20 in PEAKS Studio X+) and a requisite of a minimum unique peptide per protein for identification. For semi-quantitative analysis of proteins, the exponentially modified protein abundance index (emPAI) was calculated accordingly (Ishihama et al., 2005; Shinoda et al., 2010). Protein sequences were assessed by orthology and conserved domains against the Cluster of Orthologous Groups of proteins (COG) database using PSI-BLAST (Altschul et al., 1997) and the Conserved Domains (CD) database search (Marchler-Bauer et al., 2017), respectively. Protein location was predicted by using SignalP 5.0 Server (Almagro Armenteros et al., 2019), Transmembrane HMM (TMHMM) (Krogh et al., 2001), and pSort (Gardy et al., 2003).

### 2.6 DNA extraction and quantification of MVs

MV samples (200 μL, 35 μg of protein) were treated with DNase I (Ambion™ DNase I (RNase-free) Invitrogen™) at 37°C for 20 min, and subsequently, 450 μL of TE buffer (25:10 Tris-EDTA) were added. Lysis was induced by amendment of 50 μL of lysozyme (50 mg/mL) (Thermo Scientific), 25 μL of 5 M NaCl, and incubation at 37°C for 30 min. Then, 50 μL of 20% SDS, 5 μL of proteinase K (20 mg/mL), and 1 μL of RNase A (PureLink™ RNase A, 20 mg/mL, Invitrogen™) were added. The mixture was incubated for 1 h at 37°C, and DNA was extracted by using phenol–chloroform and isopropanol precipitation. The DNA was resuspended by adding 50 μL of nuclease-free water and quantified using the Quanti-iT™ PicoGreen dsDNA Assay Kit (Molecular Probes, Life Technologies) through an ELISA plate reader (Synergy H1, Biotek).

## 3 Results

### 3.1 Synthesis of MVs is a common trait in *Acidithiobacillaceae* family members

**Figure 1:**
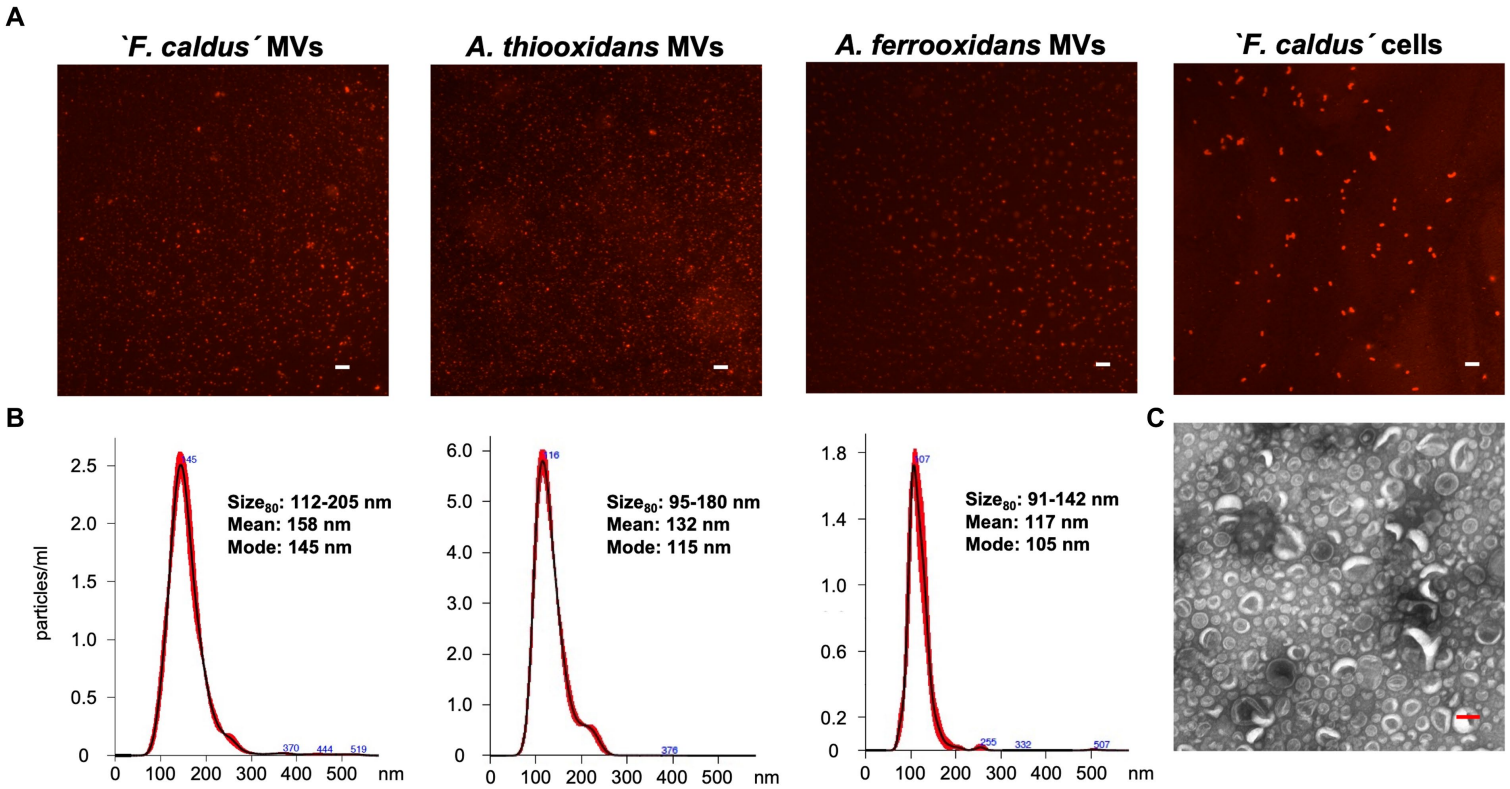
Characterization of MVs synthetized by *Acidithiobacillaceae* family members. **(A)** Visualization of MV-like particles on a glass slide. Samples obtained from ‘*F. caldus*’, *A. thiooxidans*^t^, and *A. ferrooxidans*^T^ cultures grown using sulfur as an energy source were stained with the lipid-binding dye FM 4–64 (5 μg/mL) and observed by epifluorescence microscopy. White bar, 2 μm. **(B)** Size distribution of the total population of samples of MVs using nano tracking analysis (NTA). **(C)** TEM visualization of MVs obtained from a cell-free enriched sample of ‘*F. caldus*’. Red bar, 100 nm.

To assess the potential of members of the *Acidithiobacillaceae* family to produce MVs, we selected three representative species and strains for study—‘*F. caldus*’ ATCC 51756^T^, *A. thiooxidans* ATCC 19377^T^, and *A. ferrooxidans* ATCC 23270^T^, which are the most extensively researched strains in the taxon. We obtained enriched MV fractions from elemental sulfur-grown late-exponential cultures consisting of nanosized particles that showed positive staining with the high-affinity membrane dye FM1-43 (Figure 1A). The size of the lipidic nanoparticles produced by the three species ranged between 91 and 205 nm (percentile 80) (Figure 1B) and showed a round shape (Figure 1C) in agreement with the morphology described for bacterial MVs (Nevot et al., 2006; Pérez-Cruz et al., 2015).

Given that the production of MVs has been linked to the fluidity of bacterial membranes (Kulkarni et al., 2014), which is influenced by temperature (e.g., McMahon et al., 2012), then we investigated whether heat treatment for the duration of one generation time period (8 h) could induce the production of nanosized particles in cell cultures grown under similar conditions. Using the moderate thermophilic bacterium ‘*F. caldus*’ ATCC 51756^T^ as a test case, we observed a net increase in the number of nanoparticles present in the cell-free supernatants upon heat treatment. The rate of increment was 1.6 (1.2 × 10^11^ nanoparticles) at 55°C and 2.0 (1.5 × 10^11^ nanoparticles) at 70°C, compared with the physiological condition (7.7 × 10^10^ nanoparticles at 40°C) (Supplementary Figure S1), indicating a linear relationship between vesiculation and temperature (*R*^2^ = 0.9948). Altogether, these results indicate that vesiculation is a common phenomenon in members of the *Acidithiobacillaceae* family. Thereafter, we chose ‘*F. caldus*’ as representative of the family to evaluate the effects of MVs in collective behaviors and surface colonization-related phenotypes.

### 3.2 ‘*F. caldus*’ MVs affect swarming motility in PTSM plate assays

**Figure 2:**
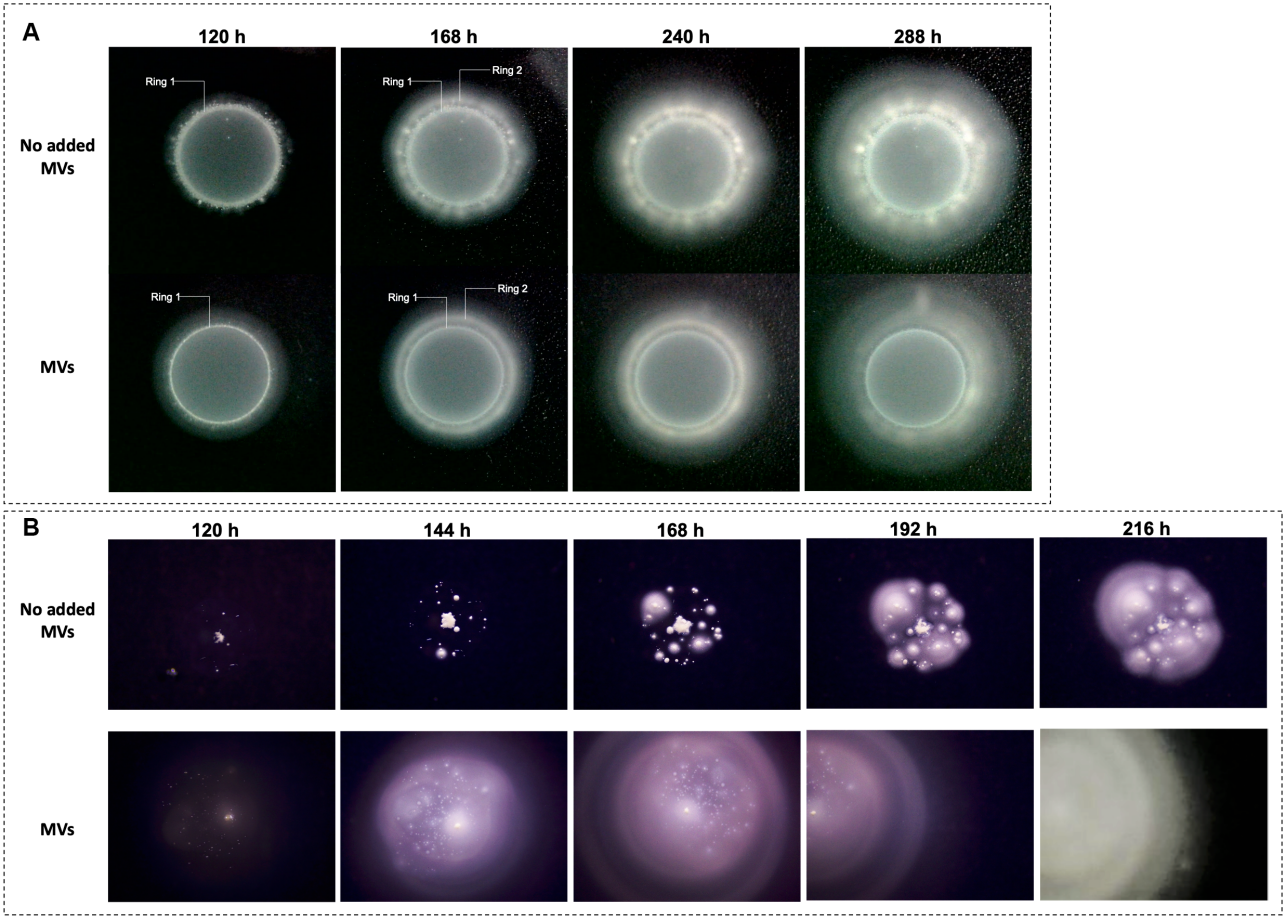
Effect of ‘*F. caldus*’ MVs on ‘*F. caldus*’ swarming motility. Two different inocula of ‘*F. caldus*’ cells were used, harvested at exponential (8 × 10^7^ cells/mL) **(A)**, and stationary phase (1.3 × 10^8^ cells/mL) **(B)**. Cells (4 × 10^9^ cells/mL) were mixed 1:1 with an MV-enriched cell-free extract (1 × 10^12^ particles/mL) or PBS buffer as a negative control; 5 μL of samples containing cells (2 × 10^7^) or cells (2 × 10^7^) plus MVs (2.5 × 10^9^ MVs) were inoculated in the center of swarming plates containing phytagel–tetrathionate semisolid medium (PTSM).

To assess whether the MVs produced by ‘*F. caldus*’ ATCC 51756^T^ could facilitate swarming, we evaluated the motility behavior of cognate cells on PTSM swarming motility plates in the presence and absence of added MVs. After 120-h incubation, control cells amended with PBS buffer only (without added MVs) accumulated at the border of the inoculation drop as evidenced by the formation of a neat white ring (Figure 2A, ring 1). At 168 h of incubation, some cells migrated from the center outward establishing microcolonies observable as discrete white dots. These microcolonies spread and coalesced, giving rise to a second ring (Figure 2A, ring 2), from where cells swarmed coordinately as revealed by the smooth concentric ring pattern developing after 10 days and onward. In contrast, cells inoculated in the presence of added MVs formed a thinner first ring (65% width of ring 1 from control cells), and then immediately started to swarm coordinately, bypassing the microcolony establishment step.

To determine whether the swarming phenotypes observed (Figure 2A) were reliant on the motility inherent to the initial cell culture, we next assessed the effect of MVs amendment on cells harvested at early stationary phase from elemental sulfur cultures, which exhibit reduced flagellar motility (see Methods). In this condition, the control cells did not migrate toward the edge of the inoculation drop, forming instead discrete colonies inside the drop area (Figure 2B). Each colony individually expanded, asymmetrically, with the side farthest from other colonies being the fastest. Colonies within this perimeter grew until coalescence, which subsequently allowed smooth swarming after 216 h (Figure 2B, last panel). In presence of added MVs, the inoculated cells spread faster and achieved smooth swarming quicker than control cells (at 144 h). These results demonstrated that amendment of MVs facilitates the swarming motility of ‘*F. caldus*’ cells regardless of the culture’s growth phase, and suggest the possibility that MVs play a role during solid surface colonization and biofilm formation.

### 3.3 MVs are structural components of acidophilic biofilms grown on sulfur

**Figure 3:**
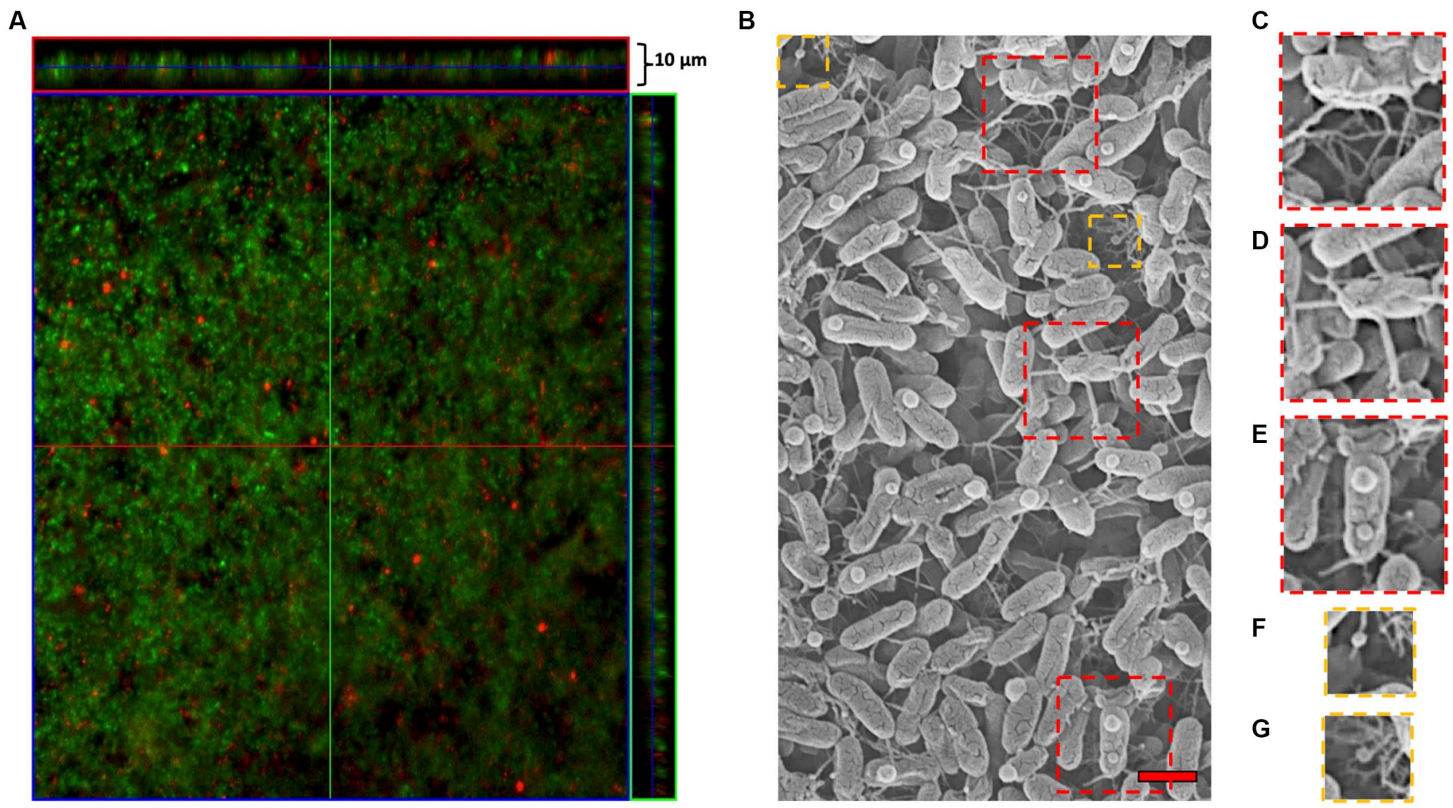
‘*F. caldus*’ biofilm on the elemental sulfur surface. **(A)** confocal microscopy using ‘live/dead’ stain (Thermo Fisher). **(B)** TEM showing MVs and tube-shaped membrane structures (TSMS). Approximately one-third of the cells in the upright layer possessed a single MV-like structure attached to its surface (37 MVs/115 cells), generally on the apical quarter of the cell. **(C)** Biofilm areas where the filaments were concentrated forming complex networks or hubs. **(D)** Multiple filaments that directly allow cell-to-cell physical interaction, communication, or exchange. **(E)** ‘*F. caldus*’ cell ‘blebbing’ produces MVs attached to surficial biofilm cells. **(F,G)** MV-TSMS association. Red bar, 1 μm.

To determine whether MVs were present in biofilms formed by ‘*F. caldus*’ ATCC 51756^T^, we incubated sulfur coupons with mid-exponential cell cultures and analyzed the established biofilms by microscopy after 72 h. CLSM confirmed the formation of mature biofilms consisting of a few layers of cells (up to 10 μm) (Figure 3A), while SEM analysis showed multiple nanotube-like filaments connecting cells within the biofilm layers (Figure 3B). We also observed biofilm areas with a higher density of filaments, some forming complex networks or hubs that seemed to connect more than two cells simultaneously (Figure 3C). Each cell in the biofilm formed between one and five filaments (Figure 3D) that physically connected it to other cells, suggesting direct cell-to-cell physical interaction, communication, or exchange. ‘*F. caldus*’ biofilms also showed the presence of spherical nanosized MV-like structures attached to surficial cells (Figure 3E). We also observed MVs associated directly with the filaments (Figures 3F,G). The co-occurrence of filaments and MVs was not observed in all samples prepared by SEM imaging (data not shown), suggesting that production of MVs in this kind of biofilms could be stochastic or that the permanence of MVs at the biofilm surface could be quite labile to handing for SEM imaging. Therefore, we decided to evaluate the presence of MVs within the biofilm structure.

**Figure 4:**
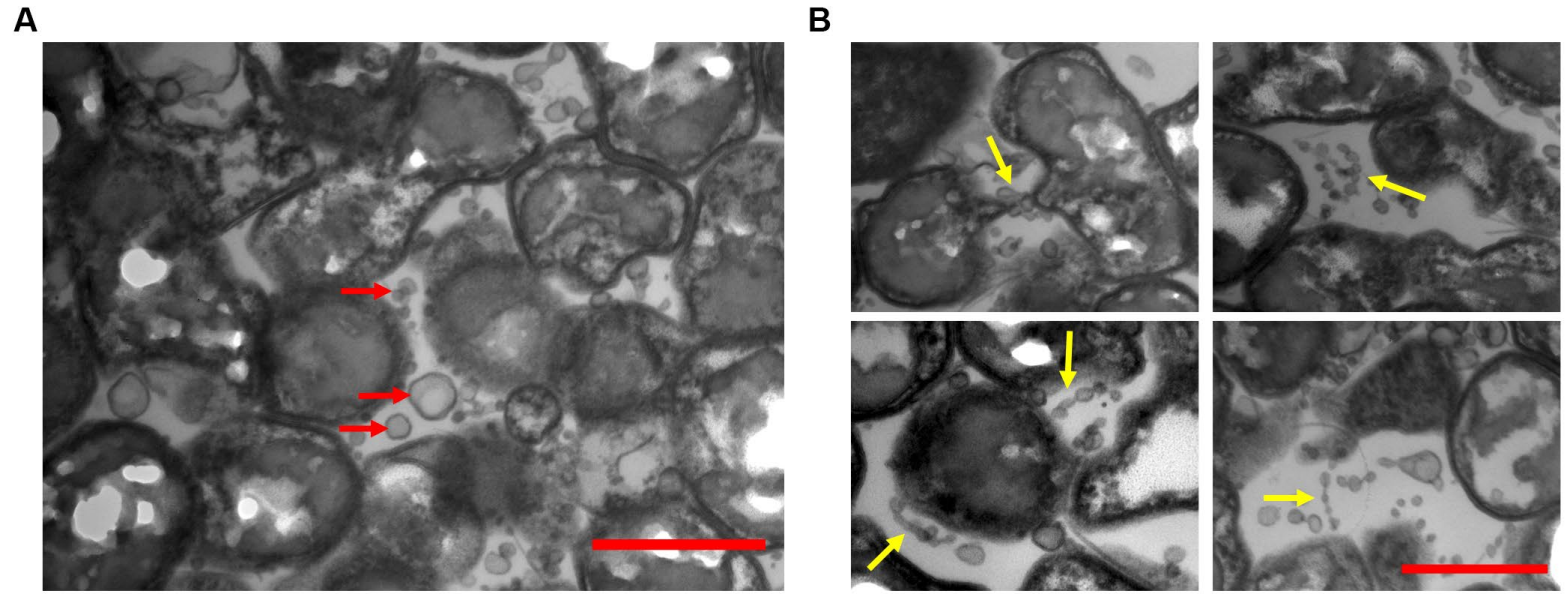
‘*F. caldus*’ biofilm on semisolid media. TEM shows thin sections of the colony biofilm formed by ‘*F. caldus*’ cells. **(A)** MVs (red arrows) and **(B)** tube-shaped membrane structures (TSMS) (yellow arrows) are depicted. Red bar, 1 μm.

**Figure 5:**
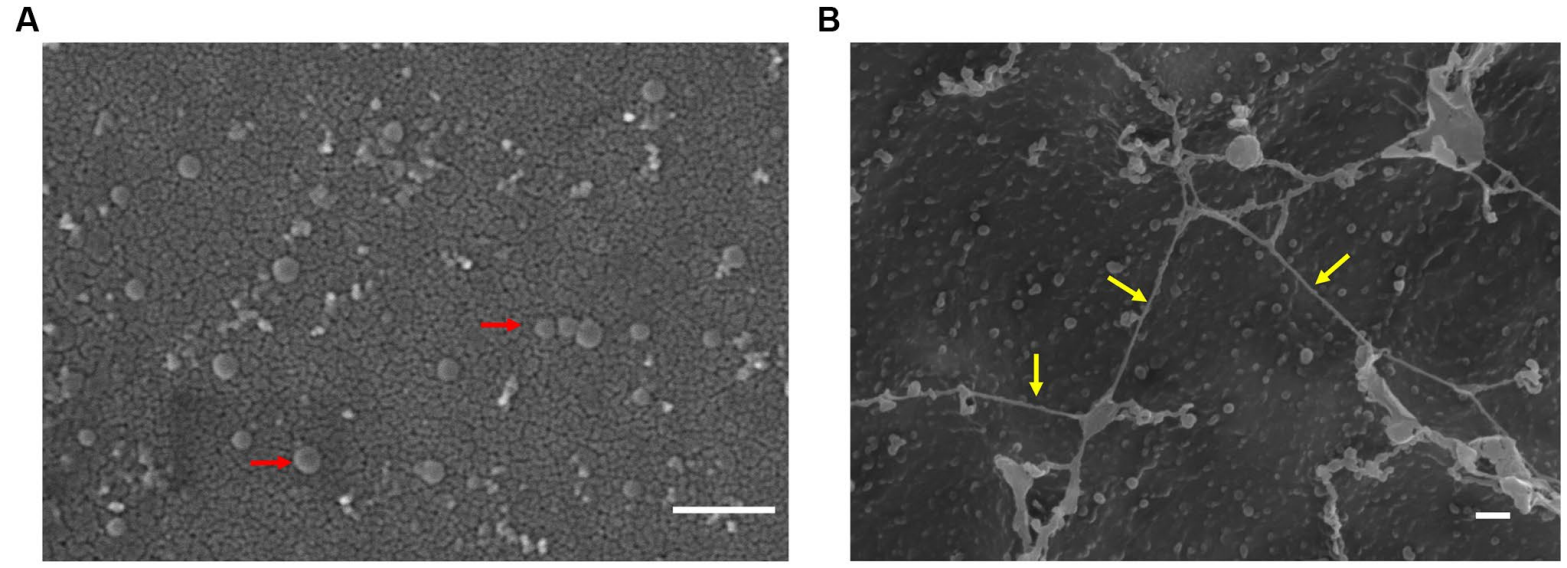
MVs enriched samples aggregate on sulfur surface. TEM of ‘*F. caldus*’ MVs on **(A)** aluminum oxide filter (Anodisc, Whatman) and **(B)** elemental sulfur. MVs (red arrows) and tube-shaped membrane structures (TSMS)-like (yellow arrows) are depicted. White bar, 1 μm.

We examined TEM thin sections (~80 nm) of a 10-day-old biofilm grown on PSTM media (0.9% phytagel), following fixation and embedding in epoxy resin. The internal structure of the biofilms formed by ‘*F. caldus*’ revealed that MVs were remarkably abundant within the intercellular space (Figure 4A). Some MVs were spatially arranged one after the other, forming chains that seemed to be partially fused (Figure 4B) and resembled the tube-shaped membranous structures (TSMSs) observed by TEM in other Gram-negative bacteria, such as *Shewanella oneidensis* and *Myxococcus xanthus*, both of which form through MVs fusion (Pirbadian et al., 2014; Remis et al., 2014; Wei et al., 2014). Incubation of ‘*F. caldus*’ MVs on sulfur coupons, in the absence of cells, showed filament structures that resemble TSMS-like occurring in mature ‘*F. caldus*’ biofilms (Figures 5A). This phenomenon was not observed when MVs were placed on aluminum oxide filters (Anodisc, Whatman) (Figure 5B), suggesting that the hydrophobic nature of sulfur could be involved in fusion promotion of MVs.

### 3.4 Amendment of MVs affects the attachment of ‘*F. caldus*’ cells to elemental sulfur

To evaluate whether MVs were promoters of biofilm formation on elemental sulfur surfaces, we explored the effects of MVs amendment on the early attachment dynamics of planktonic cells. To this end, mid-logarithmic ‘*F. caldus*’ cultures (3.3 × 10^7^ cells/mL) were exposed to elemental sulfur (powder), and cells remaining in the planktonic phase of the culture were quantified over short time periods (maximum of 3 h). As a result of cell attachment on the elemental sulfur surface, the attached cell population was observed to increase by up to 58% at 30 min and 69% after 120 min in control cultures (Table 1). This increment was less pronounced upon amendment of MVs (50% at 30 min) and more so upon addition of a 10X higher dose of MVs (15% at 30 min). These results indicate that MVs caused a net decrease in the bacterial attachment to the sulfur surface, which was proportional (a 10X difference in concentration of MVs produced a 3X decrease in cell attachment) to the concentration of MVs present in the treatment.

**Table 1:** Effect of MVs on early cell attachment on sulfur.

Time (min)	Percentage of attached cells^*^		
	No MV	MV 1X^a^	MV 10X^a^
0	0.0 ± 0.0	0.0 ± 0.0	0.0 ± 0.0
5	19.2 ± 5.4	19.2 ± 5.4	19.2 ± 5.4
15	34.6 ± 5.4	38.5 ± 0.0	11.5 ± 5.4
30	57.7 ± 5.4	50 ± 5.4	15.4 ± 10.9
45	53.8 ± 0.0	50 ± 5.4	11.5 ± 5.4
60	57.7 ± 5.4	50 ± 5.4	26.9 ± 5.4
90	65.4 ± 5.4	53.8 ± 0.0	26.9 ± 5.4
120	69.2 ± 0.0	42.3 ± 5.4	26.9 ± 5.4
180	65.4 ± 5.4	42.3 ± 5.4	26.9 ± 5.4

*Percentage of attached cells was calculated as the inverse of planktonic cells. ªLiquid media with 3% of elemental sulfur powder was incubated with or without MVs at 2 concentrations (1X = 7.5 × 10^9^ MVs and 10X = 7.5 × 10^10^ MVs) for 15 min. The treated sulfur was exposed to ‘*F. caldus*’ cells (3.25 × 10^7^ cells/mL) for 3 h.

**Figure 6:**
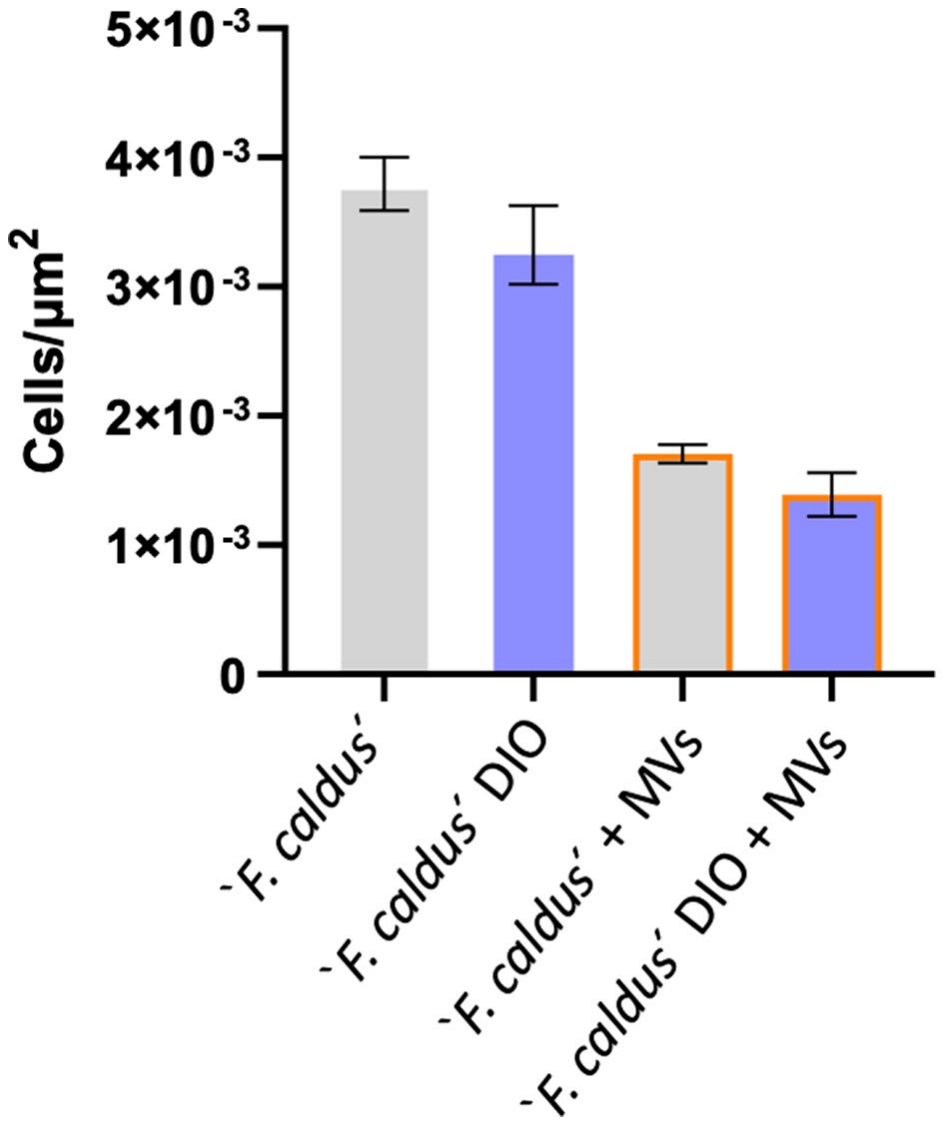
The addition of MVs impairs ‘*F. caldus*’ attachment to sulfur. ‘*F. caldus*’ was added to clean or precolonized sulfur lentils with MV and incubated as described. Cell and microcolony counts quantified with DAPI are shown. Blue bars show cells with an additional DiO staining, whereas gray bars show cells without DiO staining. Experiments with MVs addition are shown with orange borders. Bacterial attachment quantification is expressed as colonies per unit of area (μm^2^). A total of 990 images were quantified between these conditions.

Given these results, we next evaluated the effect of MVs on early attachment of ‘*F. caldus*’ cells to sulfur lentils by EFM and massive image analyses. Early cell attachment to the sulfur surfaces occurred up to 120 min, time at which growth-related or detachment effects were first evident (Supplementary Figure S2). As MVs tend to form aggregates on the lentils (Supplementary Figure S3), their discrimination from cells using fluorescent dye staining required optimization (see Methods for details). Microcolonies were observed as bright DAPI-stained cell clusters (blue channel), while MVs (green channel) appeared blurry as DiO-stained clouds (Supplementary Figure S4). Quantitative analysis of the density of attached cells and/or their microcolonies revealed differences in the number of microcolonies/μm^2^ between experimental treatments. Sulfur lentils preincubated with MVs showed a reduction in cell attachment of 42% with respect to lentils exposed to the cell suspension in the absence of MVs (adjusted *p*-value:0.002; Figure 6). Moreover, the attached cells and microcolonies exhibited minimal colocalization with the MVs as evidenced by the scarce colocalized signals of DAPI and DiO. Colocalization values of 1.2 and 3.5% were obtained with preincubation of lentils with MVs suspensions of 7.5 × 10^10^ and 7.5 × 10^9^ particles/mL, prior to the addition of cells (not shown). This suggests that colocalization of MVs and cells is unlikely to occur under our experimental settings and that MVs may competitively interfere with cell attachment to the sulfur surface, rather than recruiting cells.

### 3.5 ‘*F. caldus*’ MV-associated proteins typify vesicles as OMVs and OIMVs

**Figure 7:**
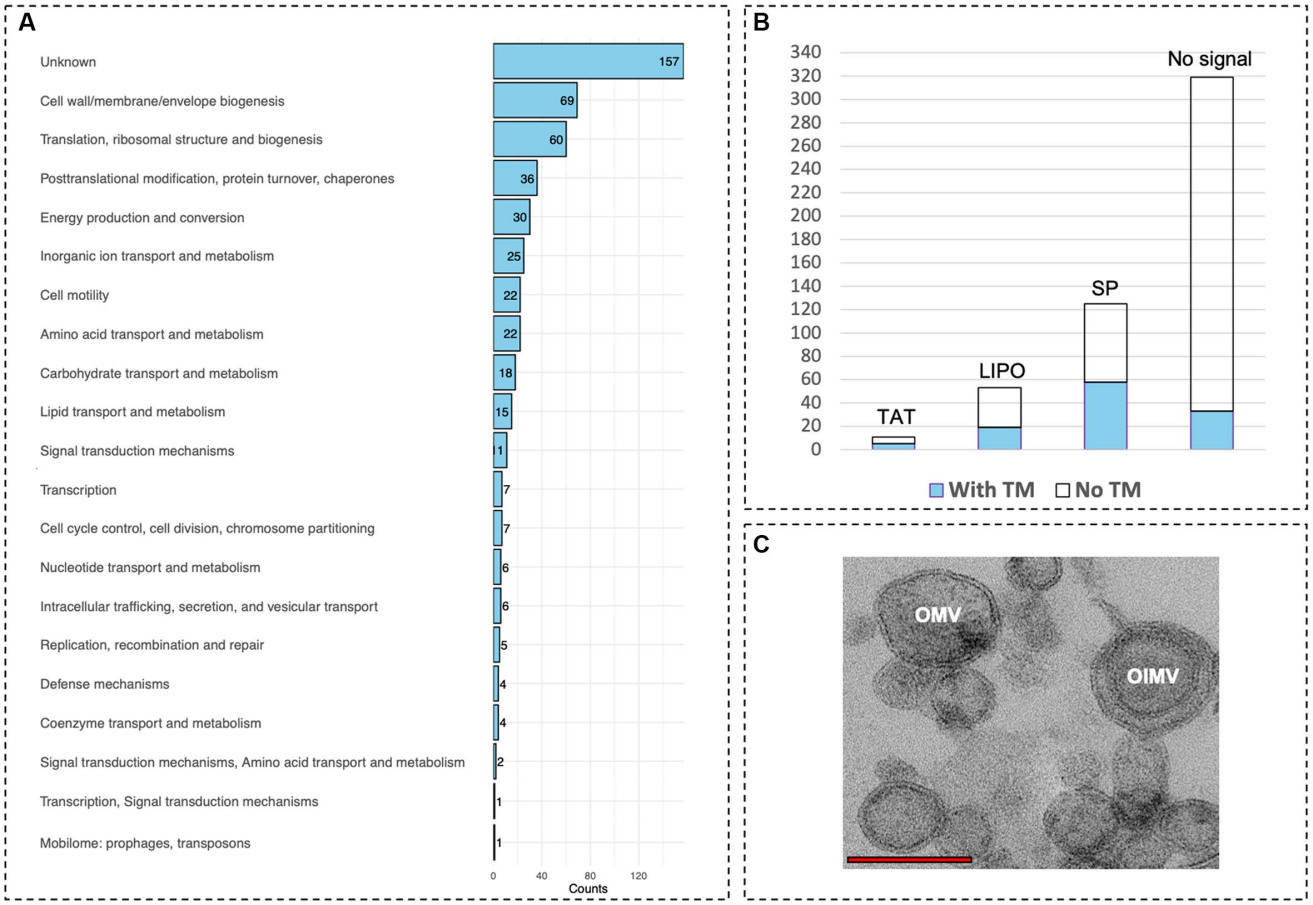
Protein content of ‘*F. caldus*’ MVs indicates the production of OMVs and OIMVs. **(A)** Proteins of MVs are shown grouped by COG functional category. **(B)** predicted subcellular location signals of proteins of MVs. **(C)** TEM showing OMVs and OIMVs. Red bar, 1 μm.

To explore the nature of the observed competitive behavior between ‘*F. caldus*’ MVs and cells upon colonization of sulfur surfaces, we assessed whether the MVs could be typified as outer membrane vesicles (OMVs) and/or as outer–inner membrane vesicles (OIMVs) (according to the definition by Toyofuku et al., 2023). For this, we characterized the protein cargo of MVs produced by sulfur-grown ‘*F. caldus*’ ATCC 51756^T^ cell cultures using LC–MS/MS proteome analysis in two independent MV batches (MV1 and MV2; Supplementary Figure S5). A total of 796 MV-associated proteins were identified in the pooled dataset, 510 of which were common between the experimental replicates, while 182 (MV1) and 104 (MV2) were exclusive to either sample. Proteins identified in each sample are shown in Supplementary Table S1. MV-associated proteins identified in the two independent assays (510/796, 64%) were annotated, classified in COG categories, and used in metabolic reconstruction analysis (Supplementary Table S1). Among the most represented COG categories recovered in the MV-associated protein fraction were those related to cell envelope (M, 10.98%), chaperones (O, 7.06%), energy metabolism (C, 4.31%), inorganic ion transport (P, 4.71%), and cell motility (N, 2.16%), known to partition between the inner membrane (IM), periplasm (P), and/or outer membrane (OM) cellular compartments (Figure 7A). Subcellular location analysis of the 510 MV-associated proteins revealed signatures of membrane targeting or destination (transmembrane domains and/or the signal peptides used by secretory, lipoprotein, or twin-arginine translocon) in 222 of the automatically annotated proteins. In addition, nine proteins without identifiable targeting signal or transmembrane segments were predicted as ‘Cytoplasmic Membrane’ proteins, and a second subset of 17 proteins with neither signal peptide nor transmembrane segments were predicted as periplasmic, outer membrane proteins, or extracellular proteins by using the PSORTb algorithm, including several flagellar-associated proteins (Figure 7B; Supplementary Table S1). Conjunctly, this indicates that nearly half of the recurrent proteins present in the MVs (248/510; 48.6%) may be targeted directly to the cell envelope.

Almost one-third of MVs proteins with no predicted signal (71/262) were related to essential processes such as replication, transcription, and translation (COG categories, L, K, and J, respectively), and these proteins are likely abundant in active cells (e.g., 46 ribosomal proteins were identified in both MVs samples). These results confirmed the presence of cytoplasmic proteins in ‘*F. caldus*’ MVs preparations, in agreement with previous studies (Choi et al., 2011; Pierson et al., 2011; Elhenawy et al., 2014; Zielke et al., 2014). In addition, the presence of intraluminal DNA, reflecting carriage by ‘*F. caldus*’ MVs of cytoplasmic content, could be confirmed by DNAse I resistant extraction and simultaneous detection of fluorescence in MV samples stained with membrane (FM4-64) and DNA (DAPI) dyes using a confocal laser scanning microscope equipped with Airyscan detection (Supplementary Figure S6). PCR amplification of both chromosomal and episomal gene markers indicated that both types of DNA could be transported by O/IMVs. The emPAI-based quantification revealed that in ‘*F. caldus*’ MVs, the proportion of cytoplasm-associated proteins (262 predicted) constituted only 20.0 mol% of the protein mass, while the predicted envelope-targeted proteins (248 in total) amounted to 79.7 mol% (Table 2; Supplementary Table S1). Among the latter, 147 proteins (61.1 mol%) were predicted to be targeted to the periplasm, OM, or extracellular space (see Supplementary Figure S7; Supplementary Table S2). Altogether, these results indicate that although OIMVs with inner membrane and cytoplasmic content are present in ‘*F. caldus*’ MVs fractions collected by our method, most of the MVs obtained can be typified as OMVs with periplasmic and outer membrane content. Accordingly, we observed MVs with one and with two membranes in slices of resin-embedded MV samples (Figure 7C), corroborating the presence of both OMVs and OIMVs in our preparations.

**Table 2:** Bioinformatic annotation of relevant and abundant proteins present in membrane vesicles produced by ‘*F. caldus.*’

locus_tag	ID	Product	MV1	MV2	emPAI_MV1 (%mol)	emPAI_MV2 (%mol)	PSORTb	Prediction	PredHel	Ranking
Type IV pilus twitching, swimming, and swarming motility										
Acaty_c1522	AIA55387.1	Type IV pilus biogenesis protein PilM	1	0	ND (ND)	ND (ND)	Cytoplasmic	OTHER	0	662
Acaty_c1523	AIA55388.1	Type IV pilus biogenesis protein PilN	1	1	0.414 (0)	0.337 (0)	Cytoplasmic membrane	OTHER	1	324
Acaty_c1524	AIA55389.1	Type IV pilus biogenesis protein PilO	1	1	ND (ND)	ND (ND)	Cytoplasmic membrane	OTHER	1	663
Acaty_c1525	AIA55390.1	Type IV pilus biogenesis protein PilP	1	1	5.32 (0.02)	3.48 (0.01)	Unknown	LIPO(Sec/SPII)	0	138
Acaty_c1526	AIA55391.1	Type IV pilus biogenesis protein PilQ	1	1	63.4 (0.18)	27.3 (0.08)	Outer membrane	SP(Sec/SPI)	0	63
Acaty_c1134	AIA55004.1	Type IV pilus biogenesis protein PilF	1	1	883 (2.56)	449 (1.3)	Outer membrane	LIPO(Sec/SPII)	1	28
Acaty_c1191	AIA55059.1	Type IV fimbrial biogenesis protein PilY1	1	1	0.443 (0)	0.578 (0)	Unknown	SP(Sec/SPI)	0	300
Acaty_c2450	AIA56294.1	Twitching motility protein PilT	1	1	ND (ND)	ND (ND)	Cytoplasmic	OTHER	0	754
Cell aggregation and substrate attachment										
Acaty_c0525	AIA54410.1	Fap amyloid fiber secretin	1	1	75.7 (0.22)	73.4 (0.21)	Cytoplasmic Membrane	SP(Sec/SPI)	0	54
Acaty_c0529	AIA54414.1	Fap protein with C39 domain	1	1	26.6 (0.08)	31.1 (0.09)	Unknown	SP(Sec/SPI)	0	73
Acaty_c0040	AIA53932.1	Extracellular matrix protein PelC	1	1	32 (0.09)	14.4 (0.04)	Outer Membrane	LIPO(Sec/SPII)	1	78
Acaty_c0036	AIA53928.1	Extracellular matrix protein PelA	1	1	13 (0.04)	10.7 (0.03)	Unknown	SP(Sec/SPI)	1	103
Acaty_c0041	AIA53933.1	Extracellular matrix protein PelD	1	1	ND (ND)	ND (ND)	Cytoplasmic	OTHER	3	515
Energy production and conversion—sulfur oxidation										
Acaty_c1580	AIA55444.1	Uptake hydrogenase small subunit precursor	1	1	ND (ND)	ND (ND)	Periplasmic	OTHER	0	673
Acaty_c1581	AIA55445.1	Uptake hydrogenase large subunit	1	1	0 (0)	0.0594 (0)	Unknown	OTHER	0	480
Acaty_c1419	AIA55285.1	Ni,Fe-hydrogenase III large subunit	0	1	ND (ND)	ND (ND)	Cytoplasmic	OTHER	0	648
Acaty_c1455	AIA55321.1	Sulfide–quinone reductase	1	1	1.39 (0)	0.926 (0)	Cytoplasmic	OTHER	0	232
Acaty_c1208	AIA55076.1	NADH dehydrogenase	1	1	1.29 (0)	0.826 (0)	Cytoplasmic	OTHER	0	240
Acaty_c2187	AIA56041.1	Glycine cleavage system H protein	1	1	9.7 (0.03)	5.63 (0.02)	Unknown	OTHER	0	121
Acaty_c2189	AIA56043.1	Glycine cleavage system H protein	1	1	0.155 (0)	0.202 (0)	Cytoplasmic	OTHER	0	387
Acaty_c2190	AIA56044.1	CoB--CoM heterodisulfide reductase subunit B	1	1	0.467 (0)	0.102 (0)	Cytoplasmic	OTHER	0	350
Acaty_c2191	AIA56045.1	CoB--CoM heterodisulfide reductase subunit C	1	1	2.76 (0.01)	3.6 (0.01)	Cytoplasmic	OTHER	0	154
Acaty_c2193	AIA56047.1	CoB--CoM heterodisulfide reductase subunit A	1	1	1.27 (0)	0.646 (0)	Unknown	OTHER	1	247
Acaty_c2194	AIA56048.1	CoB--CoM heterodisulfide reductase subunit B	1	1	0.657 (0)	0.287 (0)	Cytoplasmic	OTHER	0	308
Acaty_c2195	AIA56049.1	CoB--CoM heterodisulfide reductase subunit C	1	1	0.33 (0)	0.271 (0)	Cytoplasmic	OTHER	0	342
Acaty_c2196	AIA56050.1	NADH dehydrogenase	1	1	0.548 (0)	0.437 (0)	Cytoplasmic	OTHER	2	303
Acaty_c2197	AIA56051.1	Molybdopterin biosynthesis protein MoeB	1	1	129 (0.37)	89.6 (0.26)	Cytoplasmic	OTHER	0	50
Acaty_c2053	AIA55909.1	Sulfur oxidation protein SoxA	1	1	884 (2.56)	1,150 (3.34)	Periplasmic	SP(Sec/SPI)	0	10
Acaty_c2054	AIA55910.1	Sulfur oxidation protein SoxX	1	1	884 (2.56)	1,150 (3.34)	Unknown	OTHER	0	11
Acaty_c2057	AIA55913.1	Sulfur oxidation protein SoxB	1	1	883 (2.56)	1,150 (3.34)	Periplasmic	TAT(Tat/SPI)	0	17
Acaty_c2058	AIA55914.1	Sulfur oxidation protein SoxZ	1	1	883 (2.56)	838 (2.43)	Unknown	OTHER	0	24
Acaty_c2059	AIA55915.1	Sulfur oxidation protein SoxY	1	1	883 (2.56)	1,150 (3.34)	Unknown	SP(Sec/SPI)	0	18
Acaty_c2060	AIA55916.1	Hypothetical protein	1	1	798 (2.31)	274 (0.8)	Unknown	LIPO(Sec/SPII)	1	30
Acaty_c2208	AIA56062.1	Sulfur oxidation protein SoxX	1	1	1.86 (0.01)	0.879 (0)	Unknown	SP(Sec/SPI)	0	219
Acaty_c2209	AIA56063.1	Sulfur oxidation protein SoxY	1	1	47.7 (0.14)	33.1 (0.1)	Unknown	TAT(Tat/SPI)	1	66
Acaty_c2210	AIA56064.1	Sulfur oxidation protein SoxZ	1	1	883 (2.56)	667 (1.94)	Unknown	OTHER	0	26
Acaty_c2211	AIA56065.1	Sulfur oxidation protein SoxA	1	1	69.3 (0.2)	48.4 (0.14)	Periplasmic	SP(Sec/SPI)	0	58
Acaty_c2212	AIA56066.1	Hypothetical protein	1	1	1.98 (0.01)	2.07 (0.01)	Unknown	SP(Sec/SPI)	0	183
Acaty_c2213	AIA56067.1	Sulfur oxidation protein SoxB	1	1	19.6 (0.06)	14 (0.04)	Periplasmic	TAT(Tat/SPI)	0	86
Acaty_c1354	AIA55221.1	Hypothetical protein	1	1	195 (0.57)	79.3 (0.23)	Unknown	SP(Sec/SPI)	1	45
Membrane structure and constriction										
Acaty_c2069	AIA55925.1	Surface lipoprotein	1	1	16.1 (0.05)	18.9 (0.05)	Outer Membrane	LIPO(Sec/SPII)	0	85
Acaty_c2688	AIA56526.1	Putative ABC transporter, auxiliary component YrbC	1	1	17.8 (0.05)	9.23 (0.03)	Unknown	SP(Sec/SPI)	0	98
Acaty_c1015	AIA54887.1	Protein yceI precursor	1	1	507 (1.47)	176 (0.51)	Unknown	LIPO(Sec/SPII)	0	32
Acaty_c2070	AIA55926.1	ABC-type transport system, auxiliary component	1	1	153 (0.44)	113 (0.33)	Unknown	SP(Sec/SPI)	1	46
Acaty_c2065	AIA55921.1	hypothetical protein	1	1	884 (2.56)	1,150 (3.34)	Periplasmic	LIPO(Sec/SPII)	0	12
Acaty_c1317	AIA55184.1	Cardiolipin synthetase	1	1	38 (0.11)	15.5 (0.04)	Cytoplasmic	OTHER	0	76
Acaty_c2540	AIA56384.1	TolB protein precursor, periplasmic	1	1	883 (2.56)	1,150 (3.34)	Periplasmic	SP(Sec/SPI)	1	20
Acaty_c2653	AIA56491.1	TolB protein precursor	1	1	883 (2.56)	1,150 (3.34)	Periplasmic	SP(Sec/SPI)	1	21
Acaty_c0599	AIA54483.1	Outer membrane protein, OmpA/MotB family	1	1	884 (2.56)	1,150 (3.34)	Periplasmic	SP(Sec/SPI)	1	9
Acaty_c2613	AIA56456.1	Outer membrane protein, OmpA/MotB family	1	1	13.4 (0.04)	15.1 (0.04)	Periplasmic	LIPO(Sec/SPII)	0	93
Acaty_c0405	AIA54295.1	lipoprotein, putative	1	1	889 (2.58)	1,160 (3.37)	Unknown	LIPO(Sec/SPII)	0	1
Acaty_c2657	AIA56495.1	Outer membrane protein (OmpA/MotB precursor)	1	1	885 (2.57)	1,150 (3.34)	Outer Membrane	LIPO(Sec/SPII)	1	7
Acaty_c1096	AIA54966.1	Rare lipoprotein A precursor	1	1	13.3 (0.04)	12.1 (0.04)	Unknown	SP(Sec/SPI)	0	101
Acaty_c2533	AIA56377.1	Rare lipoprotein A precursor	1	1	2.83 (0.01)	4.43 (0.01)	Unknown	LIPO(Sec/SPII)	1	150
Acaty_c0055	AIA53947.1	Hypothetical protein	1	1	885 (2.57)	1,150 (3.34)	Periplasmic	SP(Sec/SPI)	1	4

### 3.6 MV-associated proteins play a role in substrate attachment and oxidation of RISCs

To better understand the role of MVs in ‘*F. caldus*,’ we examined the functional significance of the most abundant proteins in the MVs’ cargo, as identified by LC–MS/MS proteomic analysis, based on their mass abundance (Table 2; Supplementary Table S1). In particular, we focused on cell envelope-associated MVs protein content that could provide hints on the role played by the vesicles in substrate colonization processes. Of the extracellular structures known to play a role in collective behaviors related to twitching (type IV pilus T4P), swarming motility (flagella), cell–cell aggregation, and/or cell–surface attachment (Tight Adherence Tad pilus-like), only T4P was highly represented and abundant in the recovered MV batches analyzed. T4P MV-associated proteins included the IM alignment subcomplex PilMNOP (PilM detected only in one MV sample), the OM secretin subcomplex PilQ and PilF (pilotin), the associated adhesin PilY1, and the IM retraction ATPase PilU. Among the 50 most abundant proteins determined by emPAI (which account for 91–95% of total proteins detected) in MV1 and MV2 samples were PilQ and PilF subunits. It is known that extracellular T4P components could act as adhesins for surface interaction in several bacteria, suggesting a similar role in MVs attachment to sulfur surfaces. Flagellar proteins also occurred in both, or in either MV sample, albeit at very low relative abundances, suggesting that they are only minor or random components of the O/IMVs of ‘*F. caldus*’. Other proteins detected in the MVs, which are involved in cell–cell aggregation and biofilm formation because of their role as adhesins, were the ones forming part of the 5-protein cell envelope complex involved in the production Fap amyloid fibrils. Both the OM fiber secretin FapF and the accessory peptidase C39 FapD were abundant in the MV samples (among the 80 most abundant proteins), yet other OM components relevant to their role in adhesion (e.g., the fiber component FapC Acaty_c0530) were missing in the analyzed preparations. Additionally, cargo of MVs contains proteins involved in the synthesis of Pel exopolysaccharide. Specifically, the OM protein PelC and the periplasmic enzyme PelA were among the 100 most abundant proteins in ‘*F. caldus*’ MVs. Albeit less abundant, the inner membrane regulatory protein PelD (c-di-GMP binding) was also detected.

The protein cargo of ‘*F. caldus*’ MVs also included a broad set of enzymes involved in energy obtention and sulfur oxidation. Among these were enzymes involved in the oxidation of hydrogen (uptake hydrogenase large and small subunits and a Ni,Fe-hydrogenase III large subunit), sulfide (2 out of the 3 copies of SQR, sulfide–quinone reductases), di/persulfides (the HdrABC hetrodisulfide reductase complex and other accessory proteins encoded in the same *hdr* locus), thiosulfate (the SoxYZ, SoxAX, and SoxB components of the truncated version of the thiosulfate oxidizing multi-enzyme system, encoded in both Sox-I and Sox-II loci) and tetrathionate (the periplasmic tetrathionate hydrolase Tth), yet neither the IM-bound thiosulfate–quinone oxidoreductase (TQO) encoded in the same operon with Tth nor the cytoplasmic enzymes involved in elemental sulfur oxidation (such as SOR). Other tens of proteins related to sulfur mobilization functions (such as DsrE-like proteins, rhodanases, thioredoxins, glutaredoxins, and glutathione S-transferase) and electron transfer functions (such as the high-potential iron–sulfur protein HiPIP and cytochromes c551/c552 and P460), were also found among the MV-associated proteins (Supplementary Table S1). According to the emPAI quantitative data, SoxYZ, SoxAX, and SoxB components encoded in the Sox-I gene cluster were among the top 25 proteins in our MVs preparation, suggesting they are quantitatively important for ‘*F. caldus*’ growth during oxidation of RISCs and possibly also in MV function. Inner membrane proteins related to oxidation of RISCs were also present in the MVs proteome, albeit in lower quantities (e.g., two putative SQRs). Altogether, these results show that OMVs and OIMVs from ‘*F. caldus*’ cells grown in the presence of elemental sulfur carry OM and periplasmic components relevant to RISC oxidation. Such protein cargo could confer MVs with residual oxidation activity upon attachment to elemental sulfur particles, most likely mediated by one or several surficial adhesins also found to be relevant components of the population of MVs. This may be relevant to extend the actions of cells on the surface, by providing further oxidizing capacities for elemental sulfur and/or RISCs.

### 3.7 Highly abundant MV protein cargo controls membrane organization and constriction

Among the most abundant proteins in the LC–MS/MS proteome datasets were those related to the correct sorting, assembly, and folding of proteins at the periplasm and OM, including the complete β-barrel porins assembly machinery (BamA-E), and the periplasmic (LolA) and OM (LolB) components of Lol system for sorting and localization of lipoproteins, along with different periplasmic chaperones (peptidyl-prolyl cis-trans isomerases and proteases). Other abundant cell envelope components found were the peptidoglycan (PG)-covalently attached Braun lipoprotein (LppC), and the periplasmic (LptA) and OM (LptDE) components required for lipopolysaccharide (LPS) transport to the OM. At lower abundance, we also detected ABC transporter components involved in the trafficking of phospholipids between the IM and OM (lipid asymmetry pathway surface lipoprotein MlaA and the periplasmic protein MlaC).

Other abundant proteins found in ‘*F. caldus*’ MVs were IM proteins related to isoprenoids and lipids (hopanoids and sterols) transport and biosynthesis. These included an ortholog of *yceI* encoding the polyisoprenoid-binding protein YceI, the ABC transporter membrane protein HpnM involved in hopanoid biosynthesis, and a predicted sterol carrier protein. Although at less abundance, the cardiolipin synthetase phospholipase D was also detected in MVs. All these proteins play a role in controlling the structural organization and function of biological membranes. Congruently, several proteins involved in the maintenance of cell integrity were found among the highly abundant proteins present in ‘*F. caldus*’ MVs (top 50 most abundant proteins). These included components of Tol-Pal systems involved in the maintenance of the outer membrane and linking the IM and OM, such as TolB OM proteins, and two families of peptidoglycan-associated lipoprotein (PAL), OmpA/MotB and OprL. Also, the cell division coordinator CpoB ranked in the high abundance category. In turn, enzymes involved in PG degradation were only detected at low abundances, such as the endolytic peptidoglycan transglycosylase RlpA (involved in PG degradation in the division septum). The periplasmic component of the bacterial Cpx-two-component system, CpxP, which works along with the membrane histidine kinase CpxA and the transcriptional regulator CpxR as a global modulator of cell envelope stress in Gram-negative bacteria (including the sensing of unfolded proteins such as pilins, Zhou et al., 2011) was also found at high abundance in ‘*F. caldus*’ OMVs.

## 4 Discussion

**Figure 8:**
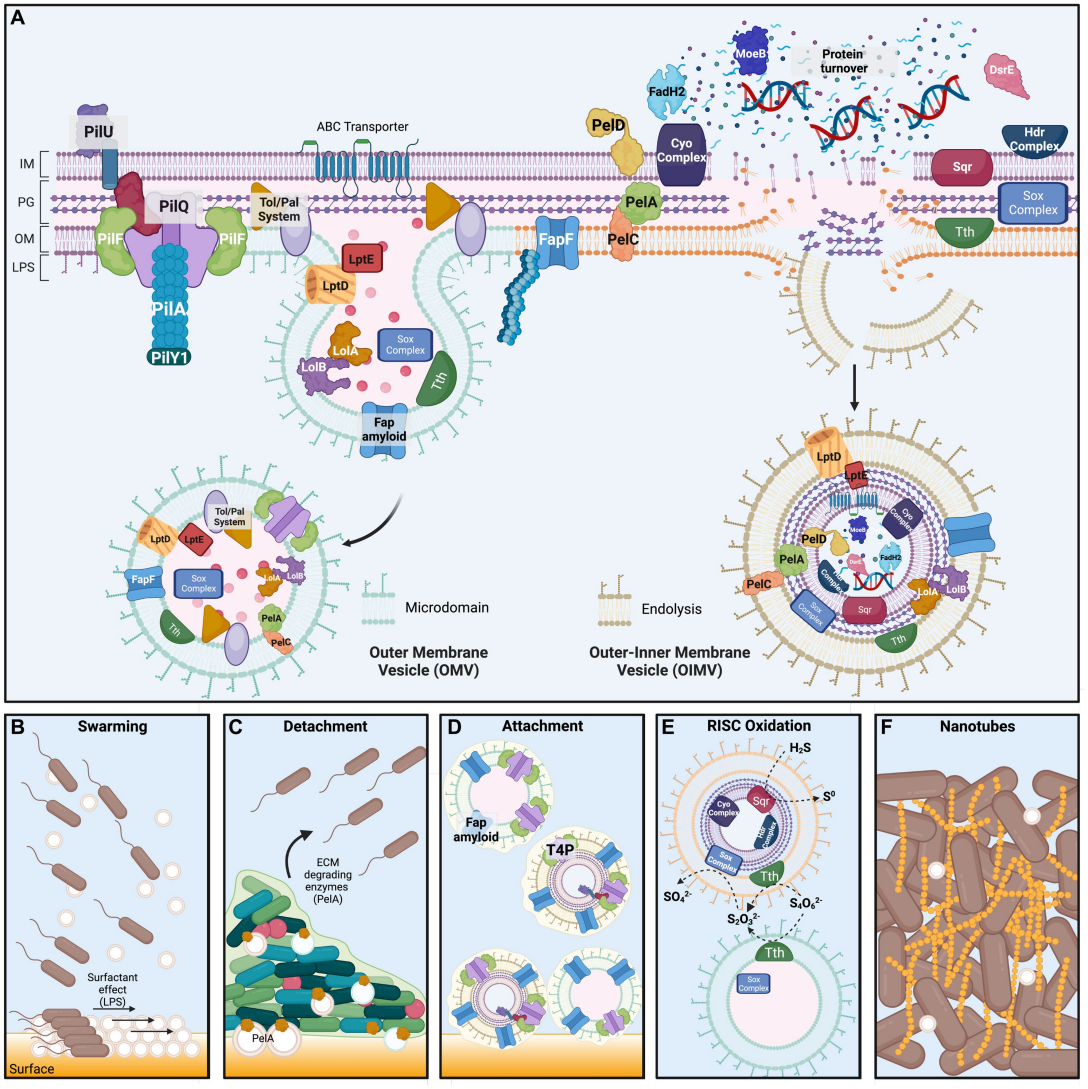
‘*F. caldus*’ Membrane Vesicles structure and proposed functions in substrate colonization. Upper panel. **(A)** Scheme of the cellular envelope of ‘*F. caldus*’ showing the most abundant proteins identified experimentally by proteomic analysis of native MV preparations. Less abundant proteins for which a physiological role is known were also included. Protein localization in the different cell compartments was inferred based on the presence of signal peptides (Sec, Tat or LipoP) and predicted transmembrane segments, along with information gathered using the PSORTb localization prediction tool (see Methods). Predicted localization and physiological roles were corroborated against published data for ‘*F. caldus*’ other *Acidithiobacillus* spp., and/or other bacterial models. Macromolecules different from proteins found to be part of the MVs cargo (e.g., DNA) are shown within MVs. The mechanisms of generation of outer membrane vesicles via ‘blebbing’ (OMVs, left) and outer–inner membrane vesicles driven by endolysins (OIMVs, right) inferred from the evidence presented in this study are also depicted. *Bottom panel*. Proposed roles for ‘*F. caldus*’ OIMVs and OMVs in **(B)** swarming motility, **(C)** attachment/detachment process, **(D)** RISC oxidation, and **(E)** tube-shaped membranous structures (TSMS) formation. For simplicity, only tetrathionate (S_4_O_6_^−2^) hydrolysis to thiosulfate (S_2_O_3_^−2^) and elemental sulfur (not shown) by Tth, and further thiosulfate oxidation to sulfate (SO_4_^−2^) by the Sox complex, are shown. Proposed roles for Fap amyloid proteins and type 4 pili during attachment of MVs to elemental sulfur are depicted, while OMV-associated PelA with a role in ECM degradation is proposed to influence biofilm detachment. Surfactant effects on swarming motility derived from surface amphipathic components (LPS) are also illustrated. The proposed role for MVs in the formation of nanotube-like TSMS is shown between cells in the final panel. Proteins and protein complexes are not drawn to scale and are shown in arbitrary order. IM, inner membrane; PG, peptidoglycan and periplasmic space; OM, outer membrane; LPS, lipopolysaccharide. Created with biorender.com.

Results presented in this study demonstrate that ‘*F. caldus*’ and other *Acidithiobacillia* class members produce MVs, which are similar in shape and size to MVs described in several other Gram-negative bacteria (Schwechheimer and Kuehn, 2015; Toyofuku et al., 2023). Composition of the MVs seems also conserved with respect to characterized bacterial MVs (e.g., OM proteins, periplasmic proteins, and LPS), consisting of lipid membranes, proteins, and frequently also DNA as evidenced by staining with specific dyes and/or purification of specific molecular fractions. The MVs collected by our enrichment method from ‘*F. caldus*’ late-exponential cell cultures grown in the presence of elemental sulfur consisted of both OMVs and OIMVs (Figure 8A), albeit at different relative abundance. Both qualitative and quantitative data support this assertion. TEM imaging of the MV preparations confirmed the presence of large quantities of single membrane MVs, accompanied by a much smaller proportion of MVs formed by two membrane bilayers, likely derived from the outer and inner membranes, representing OIMVs (Toyofuku et al., 2019). In addition, the high relative abundance and diversity of proteins predicted to localize in the periplasm, outer membrane, or extracellular space with respect to the cytoplasm recovered in the proteomic analysis further support this interpretation. In addition, only a minor proportion of the vesicles proved to carry cytoplasmic cargo, such as DNA. These types of MVs are frequently produced by bacteria, and vast variations in the proportion of OMVs:OIMVs (10:1–1:1) have been documented in the literature (Pérez-Cruz et al., 2013; Hagemann et al., 2014).

The results obtained point to blebbing as the main mechanism of MVs biogenesis in ‘*F. caldus*’ (Figure 8A) as described for other microorganisms (Toyofuku et al., 2023). Clear evidence of blebbing of MVs by biofilm-associated cells was obtained in this study, both in surficial cells observed by SEM and in cells embedded inside colony biofilms analyzed by thin sectioning and TEM. The prevailing model for bleb formation in bacteria suggests it results from the localized weakening of outer membrane—peptidoglycan (OM-PG) interactions and the associations between the outer membrane, peptidoglycan, and inner membrane (OM-PG-IM). This weakening often occurs during events such as bacterial cell division, where the inward growth of the inner membrane (IM) and peptidoglycan (PG) layer leads to a transient disruption of the septal OM-PG-IM complexes (Deatherage et al., 2009). The release of blebs during the ingrowth of the division septum was reported early on (Burdett and Murray, 1974; Weigand et al., 1976). Even if actively growing cultures of ‘*F. caldus*’ produced more MVs than late logarithmic and stationary phase cultures, supporting a certain association between production and cell division of MVs, rather than lytic bursting associated with culture decline, protrusion of MVs from the cell division septum in ‘*F. caldus*’ images was seldom observed (data not shown). Furthermore, the divisome proteins were neither well represented nor abundant in either of the MV batches produced. Also, it is well acknowledged that the OM-PG crosslinks are highly dependent on peptidoglycan remodeling processes (Cahill et al., 2015; Tian et al., 2023). Congruently, the enzymes involved in preserving a balance between the synthesis and breakdown of PG have been proposed to control OMVs synthesis. For example, the synthesis of OMVs has been attributed to the locally reduced activity of lytic transglycosylases (Lappann et al., 2013) or autolysins (endopeptidases), which allow the accumulation of peptidoglycan degradation residues, increasing the outer membrane bulging and the release of OMVs (Zhou et al., 1998; Hayashi et al., 2002). The ‘*F. caldus*’ MV-associated proteome provided hints on similar mechanisms triggering their production. The MVs from this acidophile were found to carry endopeptidases (e.g., EnvC), lytic transglycosylases (e.g., RlpA) known to play a role during cell division in model bacteria (Jorgenson et al., 2014; Cook et al., 2020), along with other key proteins related with PG remodeling during cell elongation (Fibriansah et al., 2012; Lappann et al., 2013; Alvarez et al., 2014; Morè et al., 2019), such as the D-alanyl-D-alanine carboxypeptidases DacC and MrcA, the lytic transglycosylases MltE and Slt70, and several L,D-transpeptidases.

Even if no analysis of lipids was performed in this study, evidence obtained in the epifluorescence experiments carried out with ‘*F. caldus*’ cells and MVs suggested that compositional differences between MVs and cells exist at this level. In particular, cell and MV membranes showed different affinities for the lipid-binding dye DiO. Due to its amphipathic nature, this carbocyanine dye intercalates into the lipid bilayers, with a staining behavior that is influenced by the membrane’s physical properties, being positively influenced by its fluidity (Lubart et al., 2020). Our results suggest that membranes of MVs are less ordered and less tightly packed than the rest of the cell membrane under the physicochemical conditions of our assay. Consistently, proteins related to the synthesis of polyisoprenoids, hopanoids, sterols, and phosphatidic acid, which have been linked to the formation of lipid microdomains in bacteria and/or have the ability to change membrane topology and facilitate changes in lipid bilayer curvature (Fanani and Ambroggio, 2023), were abundant in MVs. Local differences in the membrane molecular composition could promote blebbing in ‘*F. caldus*’ (Pollet et al., 2018), while the differential distribution of proteins to the lipid microdomains or surrounding these patches may induce membrane constriction and vesicle release (Szczepaniak et al., 2020). Tol/PAL proteins were highly abundant in MVs, suggesting that this may be the case.

Both microbiological assays and proteome analyses performed in this study indicate that ‘*F. caldus*’ MVs participate in processes related to cell–cell and cell–surface interactions, impacting collective behaviors such as swarming and attachment to elemental sulfur. As for other bacteria, swarming behavior in ‘*F. caldus*’ exhibited a lag phase before smooth spreading. This lag has been reported as necessary for bacterial cells to express swarming-specific functions dedicated to attracting water to the surface, reducing surface tension, and overcoming frictional forces between the bacterial cell envelope and semisolid media (Partridge and Harshey, 2013a,b). We observed that the amendment of ‘*F. caldus*’ cells with high concentrations of cognate MVs in motility plates, before the onset of swarming, facilitates initial cell spreading by smooth swarming and reduces this lag time (Figure 8B). This evidence is considered indicative of MVs affecting one or more of the swarming-specific processes. Given that the predominant fraction of the isolated vesicles were OMVs, their localization at the surface–contact interface could furnish both their inherent lipopolysaccharides (LPS) and other lipidic or lipophilic molecules that may function as surfactants. In favor of this argument, we detected several protein markers in ‘*F. caldus*’ MVs related to biosynthesis and transport of LPS, polyisoprenoids, hopanoids, and sterols, some at the high relative abundance in MVs. Yet, evidence of the presence of these compounds in the MVs was not investigated in the current study. If present, and depending on specific functional groups, the charges of these compounds could modify the amphipathic properties to ‘*F. caldus*’ OMVs and influence motility behaviors such as swarming. These observations, along with the manipulative experiments performed herein, and the natural presence of MVs in swarming cell surroundings, suggest that ‘*F. caldus*’ OMVs could act as surface-acting agents between the cells and the surface, facilitating swarming motility. In this scenario, it is likely that MVs secreted within mature biofilms could facilitate biofilm/cell detachment from sulfur surface, in concerted action with ECM degrading enzymes. The presence in ‘*F. caldus*’ MVs of PelA, which has been shown to act as a CE4 glucanase (carbohydrate esterase family 4 deacetylase and α-1,4-N-acetyl-galactosamine glycoside hydrolase; Baker et al., 2016), further supports this assertion.

Along with MVs, we observed electrodense material covering ‘*F. caldus*’ swarmer cells, indicating the presence of extracellular matrix material that could serve to draw water toward the cell to facilitate swarming. This extracellular material could be any, or a combination, of two types of exopolysaccharide potentially produced by ‘*F. caldus*’ cells: Pel polysaccharide and cellulose. Both have been recognized as key components of the extracellular matrix in diverse members of the *Acidithiobacillaceae* family (Díaz et al., 2018; Castro et al., 2020; Vera et al., 2022). Even if our protocol for enrichment of MVs probably strips the MVs of most of the extracellular polymers, it seems likely that native MVs could be surrounded by any of these. MV-specific proteomic data supported the presence of protein components of complexes dedicated to the biosynthesis of EPS, Pel, and cellulose (Figure 8B). While it is tempting to speculate that this observation relates MVs to biopolymer synthesis, no description in the current literature supports this scenario. If this was the case, only IOMVs consisting of all subcellular compartments could have the metabolic potential to exert a similar function in the vesicle, probably as residual activity. Alternatively, MVs protein cargo could be evidence of what the membrane patch that bulged out was doing in the cell (i.e., synthesizing locally biopolymers), and thus what the biopolymers linked to those MVs could be, and/or which proteins were discharged as waste material at the time of sampling the MVs. One intriguing possibility, that will require additional exploration, is that cells shed their surficial attachment arsenal through bulging MVs and thereafter detach from the biofilm for further dispersal and colonization (Figure 8C). Although our purified MV preparations were washed and probably lost the external biopolymers to some degree, the epifluorescence experiments performed showed that ‘*F. caldus*’ MVs were able to attach to the sulfur surface. According to proteome data, this could be facilitated by adhesin-like proteins contained in MVs, such as the Fap amyloid and some components of the T4P, which were among the most abundant proteins in the dataset (Figure 8D). Functional and proteomic analyses of the MVs in other bacteria have identified adhesins among relevant or primary components of their protein cargo (e.g., BabA and SabA in *Helicobacter pylori*, Olofsson et al., 2010; FadA and LktA in *Fusobacterium necrophorum*, Bista et al., 2023) supporting a general role for OMVs in mediating cell–cell and cell–surface functional interactions.

Intriguingly, our results showed that although MVs are produced by ‘*F. caldus*’ biofilms, and get attached to the sulfur surface, they do not promote biofilm development on this type of surface. In fact, clear evidence was obtained on the inverse effect, i.e., MVs prevented the initial attachment of ‘*F. caldus*’ cells to sulfur. The nature of this effect is presently unclear, yet it seems likely that it could be exerted by colonizing and blocking attachment sites for the cells. Carriage of the same adhesins by OMVs and cells has been invoked previously as a cause for the negative effect in bacterium–host interactions due to competitive effects (Olofsson et al., 2010). We investigated the functional repertoire of MV proteins to seek evidence for an alternative explanation to the observed negative effect. For instance, cells and MVs could compete for sulfur sites due to the presence of alike sulfur oxidation functions (Figure 8E). Indeed, many proteins involved in RISCs oxidation were present in the MVs, the vast majority of which are known or predicted be located in the OM and periplasmic space. While these findings support the intriguing hypothesis of cell-independent MV-driven RISCs oxidation, no demonstration of this was achieved in the present study. Further research is required to explore such potential of MVs, along with understanding the underlying mechanisms and implications.

Although the dynamics of biofilm formation by ‘*F. caldus*’ biofilms formation on elemental sulfur has been well characterized (Castro et al., 2015, 2020), the role of membrane vesicles (MVs) in this process represents a novel aspect of the study. In this study, we demonstrated that MVs are a highly abundant component of ‘*F. caldus*’ biofilm ECMs, which exist as individual entities on the surface of cells and in the intercellular space between cells within biofilms, yet also as chains of MVs (Figure 8F). These chains could eventually represent thin sections of nanotubes or TSMS, whose nature and role have remained mostly untapped. Other Gram-negative bacteria such as *Shewanella oneidensis* and *Myxococcus xanthus* form TSMSs through fusion of MVs (Pirbadian et al., 2014; Remis et al., 2014; Wei et al., 2014); meanwhile, cryo-electron microscopy of *Vibrio vulnificus* cultures have shown that individual MVs are pinched off from TSMSs in a ‘beads-on-a-string’ fashion (Hampton et al., 2017). Although it is not clear whether the primary function of ‘*F. caldus*’ OM blebbing is to form TSMS, we have observed the formation of TSMS-like derived from MVs extracts on the elemental sulfur surface as well as from apparently fused MVs. Thus, for the first time, this study suggests the membrane nature of the filaments reported in biofilms developed by the acidithiobacilli. This implies that these cell connections are projections of the cell envelope, which may contain proteins that work at the OM and/or at the periplasm. This could favor cell–cell interactions and molecular exchange of acid-labile compounds and molecules under the harsh conditions of the ‘*F. caldus*’ habitat. The presumed envelope nature of the acidithiobacilli filaments also implies that cells may share key proteins found in MVs, such as those involved in energy obtention and electron transport, resembling nanowires described in *Shewanella* or *Myxococcus* spp. (Pirbadian et al., 2014; Remis et al., 2014). In addition, or alternatively, the TSMS could play a structural role in cell–cell interactions during biofilm formation and detachment cycles. Further study is deemed necessary to clarify these aspects.

## 5 Conclusion

‘*F. caldus*’ and at least two other *Acidithiobacillaceae* family members produce MVs under planktonic and substrate-attached growth conditions as confirmed by qualitative data from epifluorescence, transmission and scanning electron microscopy, and quantitative data from nano-tracking analyses. ‘*F. caldus*’ MVs were mainly classified as outer membrane vesicles (OMVs) based on visualization of their structure and proteomic analyses of purified MVs, yet outer–inner membrane vesicles (OIMVs) were also detected in our preparations. Blebbing seems to be the principal mechanism for synthesis of MVs in this bacterium, probably favored by local differentiation of lipidic microdomains and concurrence of membrane remodeling proteins in such domains. The MVs proved to be structural components of the biofilms formed by ‘*F. caldus*’ in both sulfur surfaces and tetrathionate semi-solid media plates, where they were abundant at the surface and intercellularly. Some of these MVs appeared to fuse, forming chain-like structures resembling tube-shaped membranous structures observed in other bacteria. Vesiculation of ‘*F. caldus*’ cells increased with heat stress, indicating that temperature and possibly other physicochemical treatments affecting the cell envelope integrity and/or the fluidity of the membrane, promote MVs production. ‘*F. caldus*’ cells exposed to amendment of MVs displayed enhanced swarming motility in PTSM plates, where MVs played a role in facilitating the initiation of swarming with respect to control treatments, probably acting as surface-active agents or surfactants. Also, the native MVs produced by ‘*F. caldus*,’ which proved to have the intrinsic capacity of attachment to sulfur surfaces, reduced early attachment of planktonic cells to sulfur and negatively affected biofilm development on sulfur lentils as revealed by epifluorescence microscopy. The observed interference could be caused by occupancy of MVs of the sulfur sites available for cell attachment, pointing to similar mechanisms of attachment for both entities. This was strongly supported by epifluorescence colocalization experiments, which showed low interaction between OMVs and cells, and by proteomic data, which showed enrichment in ‘*F. caldus*’ MVs of proteins linked to the synthesis of exopolymers, adhesins (Fap amyloid and T4P), and OM proteins related to the oxidation of RISCs. Altogether, these results indicate that independent of the lifestyle (planktonic versus attached), the production of MVs by ‘*F. caldus*’ is quantitatively significant and plays relevant roles in both cell–cell and cell–surface interactions, broadening the cell boundaries to the intercellular space and colonizable mineral surfaces that are key to *Acidithiobacillia* class bacteria ecophysiology.

## Data availability statement

The data presented in the study are deposited in the ProteomeXchange Consortium via the PRIDE repository (Perez-Riverol et al., 2022), accession number PXD04744.

## Author contributions

SR: Data curation, Formal analysis, Investigation, Methodology, Validation, Visualization, Writing – original draft. SB: Data curation, Funding acquisition, Investigation, Project administration, Software, Visualization, Writing – review & editing. MS-B: Methodology, Validation, Writing – original draft. JD-R: Software, Visualization, Writing – review & editing. FKO: Methodology, Validation, Writing – original draft. MV-G: Methodology, Resources, Writing – original draft. PM-B: Conceptualization, Resources, Writing – original draft. MV: Investigation, Resources, Writing – review & editing. RQ: Conceptualization, Formal analysis, Funding acquisition, Investigation, Project administration, Visualization, Writing – original draft, Writing – review & editing. MC: Conceptualization, Formal analysis, Funding acquisition, Investigation, Methodology, Project administration, Supervision, Validation, Visualization, Writing – original draft, Writing – review & editing.

## Footnotes

^1^ http://rsbweb.nih.gov/ij/

## Glossary

ARD/AMD: acid rock/mine drainage
CLSM: confocal laser scanning microscopy
CDD: conserved domains database
c-di-GMP: cyclic diguanylate
COG: Cluster of Orthologous Groups of proteins
DAPI, 4′: 6-diamidino-2-phenylindole
ECM: extracellular matrix
emPAI: exponentially modified protein abundance index
EPS: extracellular polymeric substances
eDNA: extracellular DNA
Hdr: hetrodisulfide reductase
HiPIP: high-potential iron–sulfur protein
LC–MS/MS: liquid chromatography–tandem mass spectrometry
LPS: lipopolysaccharide
IM: inner membrane
MV: membrane vesicle
OIMV: outer–inner membrane vesicles
OM: outer membrane
OMV: outer membrane vesicles
PG: peptidoglycan
PBS: Phosphate-buffered saline
PTSM: phytagel–tetrathionate semisolid medium
RISC: reduced inorganic sulfur compound
SEM: scanning electron microscopy
SOR: sulfur oxygenase reductase
SQR: Sulfide:quinone reductase
TEM: transmission electron microscopy
TMHMM: transmembrane HMM
TQO: thiosulfate–quinone oxidoreductase
TTH: tetrathionate hydrolase
TSMS: tube-shaped membranous structure

## References

[ref1] Almagro Armenteros J. J. Tsirigos K. D. Sønderby C. K. Petersen T. N. Winther O. Brunak S.. (2019). SignalP 5.0 improves signal peptide predictions using deep neural networks. Nat. Biotechnol. 37, 420–423. doi: 10.1038/s41587-019-0036-z 30778233

[ref2] Altschul S. F. Madden T. L. Schäffer A. A. Zhang J. Zhang Z. Miller W.. (1997). Gapped BLAST and PSI-BLAST: a new generation of protein database search programs. Nucleic Acids Res. 25, 3389–33402. doi: 10.1093/nar/25.17.3389 9254694 PMC146917

[ref3] Alvarez L. Espaillat A. Hermoso J. A. de Pedro M. A. Cava F. (2014). Peptidoglycan remodeling by the coordinated action of multispecific enzymes. Microb. Drug Resist. 20, 190–198. doi: 10.1089/mdr.2014.0047 24799190 PMC4050494

[ref4] Baker P. Hill P. J. Snarr B. D. Alnabelseya N. Pestrak M. J. Lee M. J.. (2016). Exopolysaccharide biosynthetic glycoside hydrolases can be utilized to disrupt and prevent *Pseudomonas aeruginosa* biofilms. Sci. Adv. 2:e150163. doi: 10.1126/sciadv.1501632 PMC4928890 27386527

[ref5] Baumgarten T. Sperling S. Seifert J. Von Bergen M. Steiniger F. Wick L. Y.. (2012). Membrane vesicle formation as a multiple-stress response mechanism enhances *Pseudomonas putida* DOT-T1E cell surface hydrophobicity and biofilm formation. Appl. Environ. Microbiol. 78, 6217–6224. doi: 10.1128/AEM.01525-12 22752175 PMC3416621

[ref6] Bellenberg S. Díaz M. Nanni N. Sand W. Poetsch A. Guiliani N.. (2014). Biofilm formation, communication and interactions of leaching bacteria during colonization of pyrite and sulfur surfaces. Res. Microbiol. 165, 773–781. doi: 10.1016/j.resmic.2014.08.006 25172572

[ref7] Bista P. K. Pillai D. Narayanan S. K. (2023). Outer-membrane vesicles of *Fusobacterium necrophorum*: a proteomic, lipidomic, and functional characterization. Microorganisms 11:2082. doi: 10.3390/microorganisms11082082 37630642 PMC10458137

[ref8] Bonnington K. E. Kuehn M. J. (2014). Protein selection and export via outer membrane vesicles. Biochim. Biophys. Acta 1843, 1612–1619. doi: 10.1016/j.bbamcr.2013.12.011 24370777 PMC4317292

[ref9] Burdett I. D. J. Murray R. G. E. (1974). Electron microscope study of septum formation in *Escherichia coli* strains B and B-r during synchronous growth. J. Bacteriol. 119, 1039–1056. doi: 10.1128/jb.119.3.1039-1056.1974 4604418 PMC245711

[ref10] Busatto S. Vilanilam G. Ticer T. Lin W. L. Dickson D. W. Shapiro S.. (2018). Tangential flow filtration for highly efficient concentration of extracellular vesicles from large volumes of fluid. Cells 7:273. doi: 10.3390/cells7120273 30558352 PMC6315734

[ref11] Cahill B. K. Seeley K. W. Gutel D. Ellis T. N. (2015). *Klebsiella pneumoniae* O antigen loss alters the outer membrane protein composition and the selective packaging of proteins into secreted outer membrane vesicles. Microbiol. Res. 180, 1–10. doi: 10.1016/j.micres.2015.06.012 26505306

[ref12] Castro M. Deane S. Ruiz L. Rawlings D. E. Guiliani N. (2015). Diguanylate cyclase null mutant reveals that C-Di-GMP pathway regulates the motility and adherence of the extremophile bacterium *Acidithiobacillus caldus*. PLoS One 10:e0116399. doi: 10.1371/journal.pone.0116399 25689133 PMC4331095

[ref13] Castro M. Díaz M. Moya-Beltrán A. Guiliani N. (2020). “Cyclic di-GMP signaling in extreme acidophilic Bacteria” in Microbial cyclic di-nucleotide signaling. eds. Chou S. H. Guiliani N. Lee V. Römling U. (Cham: Springer International Publishing), 337–353.

[ref14] Chattopadhyay M. K. Jaganandham M. V. (2015). Vesicles-mediated resistance to antibiotics in bacteria. Front. Microbiol. 6:758. doi: 10.3389/fmicb.2015.00758 26257725 PMC4511839

[ref15] Choi D. S. Kim D. K. Choi S. J. Lee J. Choi J. P. Rho S.. (2011). Proteomic analysis of outer membrane vesicles derived from *Pseudomonas aeruginosa*. Proteomics 11, 3424–3429. doi: 10.1002/pmic.201000212 21751344

[ref16] Chutkan H. Macdonald I. Manning A. Kuehn M. J. (2013). Quantitative and qualitative preparations of bacterial outer membrane vesicles. Methods Mol. Biol. 966, 259–272. doi: 10.1007/978-1-62703-245-2_16 23299740 PMC4317262

[ref17] Cook J. Baverstock T. C. McAndrew M. B. L. Stansfeld P. J. Roper D. I. Crow A. (2020). Insights into bacterial cell division from a structure of EnvC bound to the FtsX periplasmic domain. Proc. Natl. Acad. Sci. U. S. A. 117, 28355–28365. doi: 10.1073/pnas.2017134117 33097670 PMC7668044

[ref18] Covarrubias P. C. Moya-Beltrán A. Atavales J. Moya-Flores F. Tapia P. S. Acuña L. G.. (2018). Occurrence, integrity, and functionality of AcaML1–like viruses infecting extreme acidophiles of the *Acidithiobacillus* species complex. Res. Microbiol. 169, 628–637. doi: 10.1016/j.resmic.2018.07.005 30138723

[ref19] Covarrubias P. C. Muñoz R. Bobadilla-Fazzini R. Martinez P. Quatrini R. (2017). Are there viruses in industrial bioleaching econiches? Solid State Phenom. 262, 521–525. doi: 10.4028/www.scientific.net/SSP.262.521

[ref20] Deatherage B. L. Cooksona B. T. (2012). Membrane vesicle release in bacteria, eukaryotes, and archaea: a conserved yet underappreciated aspect of microbial life. Infect. Immun. 80, 1948–1957. doi: 10.1128/IAI.06014-11 22409932 PMC3370574

[ref21] Deatherage B. L. Lara J. C. Bergsbaken T. Barrett S. L. R. Lara S. Cookson B. T. (2009). Biogenesis of bacterial membrane vesicles. Mol. Microbiol. 72, 1395–1407. doi: 10.1111/j.1365-2958.2009.06731.x 19432795 PMC2745257

[ref22] Díaz M. Castro M. Copaja S. Guiliani N. (2018). Biofilm formation by the acidophile bacterium *Acidithiobacillus thiooxidans* involves c-di-GMP pathway and pel exopolysaccharide. Genes 9:113. doi: 10.3390/genes9020113 29466318 PMC5852609

[ref23] Díaz M. San Martin D. Castro M. Vera M. Guiliani N. (2021). Quorum sensing signaling molecules positively regulate c-di-GMP effector PelD encoding gene and PEL exopolysaccharide biosynthesis in extremophile bacterium *Acidithiobacillus thiooxidans*. Genes 12:69. doi: 10.3390/genes12010069 33430222 PMC7825692

[ref24] Domingues S. Nielsen K. M. (2017). Membrane vesicles and horizontal gene transfer in prokaryotes. Curr. Opin. Microbiol. 22, 16–21. doi: 10.1016/j.mib.2017.03.012 28441577

[ref25] Edwards K. J. Bond P. L. Banfield J. F. (2000). Characteristics of attachment and growth of *Thiobacillus caldus* on sulphide minerals: a chemotactic response to sulphur minerals? Environ. Microbiol. 2, 324–332. doi: 10.1046/j.1462-2920.2000.00111.x 11200434

[ref26] Elhenawy W. Debelyy M. O. Feldman M. F. (2014). Preferential packing of acidic glycosidases and proteases into *Bacteroides* outer membrane vesicles. MBio 5:e00909-14. doi: 10.1128/mBio.00909-14 24618254 PMC3952158

[ref27] Fanani M. L. Ambroggio E. E. (2023). Phospholipases and membrane curvature: what is happening at the surface? Membranes 13:190. doi: 10.3390/membranes13020190 36837693 PMC9965983

[ref28] Fibriansah G. Gliubich F. I. Thunnissen A. M. W. H. (2012). On the mechanism of peptidoglycan binding and cleavage by the endo-specific lytic transglycosylase MltE from *Escherichia coli*. Biochemistry 51, 9164–9177. doi: 10.1021/bi300900t 23075328

[ref29] Gardy J. L. Spencer C. Wang K. Ester M. Tusnády G. E. Simon I.. (2003). PSORT-B: improving protein subcellular localization prediction for gram-negative bacteria. Nucleic Acids Res. 31, 3613–3617. doi: 10.1093/nar/gkg602 12824378 PMC169008

[ref30] Gehrke T. Telegdi J. Thierry D. Sand W. (1998). Importance of extracellular polymeric substances from *Thiobacillus ferrooxidans* for bioleaching. Appl. Environ. Microbiol. 64, 2743–2747. doi: 10.1128/aem.64.7.2743-2747.1998 9647862 PMC106458

[ref31] Gerritzen M. J. H. Salverda M. L. M. Martens D. E. Wijffels R. H. Stork M. (2019). Spontaneously released *Neisseria meningitidis* outer membrane vesicles as vaccine platform: production and purification. Vaccine 37, 6978–6986. doi: 10.1016/j.vaccine.2019.01.076 31383485

[ref32] Gill S. Catchpole R. Forterre P. (2019). Extracellular membrane vesicles in the three domains of life and beyond. FEMS Microbiol. Rev. 43, 273–303. doi: 10.1093/femsre/fuy042 30476045 PMC6524685

[ref33] González D. M. Lara R. H. Alvarado K. N. Valdez-Perez D. Navarro-Contreras H. R. Cruz R.. (2012). Evolution of biofilms during the colonization process of pyrite by *Acidithiobacillus thiooxidans*. Appl. Microbiol. Biotechnol. 93, 763–775. doi: 10.1007/s00253-011-3465-2 21773763

[ref34] Hagemann S. Stöger L. Kappelmann M. Hassl I. Ellinger A. Velimirov B. (2014). DNA-bearing membrane vesicles produced by *Ahrensia kielensis* and *Pseudoalteromonas marina*. J. Basic Microbiol. 54, 1062–1072. doi: 10.1002/jobm.201300376 24136405

[ref35] Hampton C. M. Guerrero-Ferreira R. C. Storms R. E. Taylor J. V. Yi H. Gulig P. A.. (2017). The opportunistic pathogen *Vibrio vulnificus* produces outer membrane vesicles in a spatially distinct manner related to capsular polysaccharide. Front. Microbiol. 8:2177. doi: 10.3389/fmicb.2017.02177 29163452 PMC5681939

[ref36] Harneit K. Göksel A. Kock D. Klock J. H. Gehrke T. Sand W. (2006). Adhesion to metal sulfide surfaces by cells of *Acidithiobacillus ferrooxidans, Acidithiobacillus thiooxidans* and *Leptospirillum ferrooxidans*. Hydrometallurgy 83, 245–254. doi: 10.1016/j.hydromet.2006.03.044

[ref37] Haurat M. F. Elhenawy W. Feldman M. F. (2015). Prokaryotic membrane vesicles: new insights on biogenesis and biological roles. Biol. Chem. 396, 95–109. doi: 10.1515/hsz-2014-0183 25178905

[ref38] Hayashi J. Hamada N. Kuramitsu H. K. (2002). The autolysin of *Porphyromonas gingivalis* is involved in outer membrane vesicle release. FEMS Microbiol. Lett. 216, 217–222. doi: 10.1111/j.1574-6968.2002.tb11438.x 12435505

[ref39] Hedrich S. Schippers A. (2021). Distribution of Acidophilic Microorganisms in Natural and Man-made Acidic Environments. Curr. Issues. Mol. Biol. 40, 25–48. doi: 10.21775/cimb.040.025 32159522

[ref40] Ishihama Y. Oda Y. Tabata T. Sato T. Nagasu T. Rappsilber J.. (2005). Exponentially modified protein abundance index (emPAI) for estimation of absolute protein amount in proteomics by the number of sequenced peptides per protein. Mol. Cell. Proteomics 4, 1265–1272. doi: 10.1074/mcp.M500061-MCP200 15958392

[ref41] Johnson D. B. (2014). Biomining-biotechnologies for extracting and recovering metals from ores and waste materials. Curr. Opin. Biotechnol. 30, 24–31. doi: 10.1016/j.copbio.2014.04.008 24794631

[ref42] Jones G. E. Starkey R. L. (1961). Surface-active substances produced by *Thiobacillus thiooxidans*. J. Bacteriol. 82, 788–789. doi: 10.1128/jb.82.5.788-789.1961 14452261 PMC279252

[ref43] Jorgenson M. A. Chen Y. Yahashiri A. Popham D. L. Weiss D. S. (2014). The bacterial septal ring protein RlpA is a lytic transglycosylase that contributes to rod shape and daughter cell separation in *Pseudomonas aeruginosa*. Mol. Microbiol. 93, 113–128. doi: 10.1111/mmi.12643 24806796 PMC4086221

[ref44] Kadurugamuwa J. L. Beveridge T. J. (1996). Bacteriolytic effect of membrane vesicles from *Pseudomonas aeruginosa* on other bacteria including pathogens: conceptually new antibiotics. J. Bacteriol. 178, 2767–2774. doi: 10.1128/jb.178.10.2767-2774.1996 8631663 PMC178010

[ref45] Kearns D. B. (2010). A field guide to bacterial swarming motility. Nat. Rev. Microbiol. 8, 634–644. doi: 10.1038/nrmicro2405 20694026 PMC3135019

[ref46] Klimentová J. Stulík J. (2015). Methods of isolation and purification of outer membrane vesicles from gram-negative bacteria. Microbiol. Res. 170, 1–9. doi: 10.1016/j.micres.2014.09.006 25458555

[ref47] Knickerbocker C. Nordstrom D. K. Southam G. (2000). The role of ‘blebbing’ in overcoming the hydrophobic barrier during biooxidation of elemental sulfur by *Thiobacillus thiooxidans*. Chem. Geol. 169, 425–433. doi: 10.1016/S0009-2541(00)00221-7

[ref48] Krogh A. Larsson B. von Heijine G. Sonnhammer E. L. (2001). Predicting transmembrane protein topology with a hidden Markov model: application to complete genomes. J. Mol. Biol. 305, 567–580. doi: 10.1006/jmbi.2000.4315 11152613

[ref49] Kuehn M. J. Kesty N. C. (2005). Bacterial outer membrane vesicles and the host-pathogen interaction. Genes Dev. 19, 2645–2655. doi: 10.1101/gad.1299905 16291643

[ref50] Kulkarni H. M. Swamy C. V. B. Jagannadham M. V. (2014). Molecular characterization and functional analysis of outer membrane vesicles from the Antarctic bacterium *Pseudomonas syringae* suggest a possible response to environmental conditions. J. Proteome Res. 13, 1345–1358. doi: 10.1021/pr4009223 24437924

[ref51] Lappann M. Otto A. Becher D. Vogel U. (2013). Comparative proteome analysis of spontaneous outer membrane vesicles and purified outer membranes of *Neisseria meningitidis*. J. Bacteriol. 195, 4425–4435. doi: 10.1128/JB.00625-13 23893116 PMC3807460

[ref52] Liu J. Cvirkaite-Krupovic V. Commere P. H. Yang Y. Zhou F. Forterre P.. (2021). Archaeal extracellular vesicles are produced in an ESCRT-dependent manner and promote gene transfer and nutrient cycling in extreme environments. ISME J. 15, 2892–2905. doi: 10.1038/s41396-021-00984-0 33903726 PMC8443754

[ref53] Liu J. Soler N. Gorlas A. Cvirkaite-Krupovic V. Krupovic M. Forterre P. (2021). Extracellular membrane vesicles and nanotubes in Archaea. Microlife 2:uqab007. doi: 10.1093/femsml/uqab007 37223257 PMC10117752

[ref54] Lubart Q. Hannestad J. K. Pace H. Fjällborg D. Westerlund F. Esbjörner E. K.. (2020). Lipid vesicle composition influences the incorporation and fluorescence properties of the lipophilic sulphonated carbocyanine dye SP-DiO. Phys. Chem. Chem. Phys. 22, 8781–8790. doi: 10.1039/c9cp04158c 32285050

[ref55] MacDonald I. A. Kuehn M. J. (2012). Offense and defense: microbial membrane vesicles play both ways. Res. Microbiol. 163, 607–618. doi: 10.1016/j.resmic.2012.10.020 23123555 PMC3518640

[ref56] Manning A. J. Kuehn M. J. (2011). Contribution of bacterial outer membrane vesicles to innate bacterial defense. BMC Microbiol. 11:258. doi: 10.1186/1471-2180-11-258 22133164 PMC3248377

[ref57] Marchler-Bauer A. Bo Y. Han L. He J. Lanczycki C. J. Lu S.. (2017). CDD/SPARCLE: functional classification of proteins via subfamily domain architectures. Nucleic Acids Res. 45, D200–D203. doi: 10.1093/nar/gkw1129 27899674 PMC5210587

[ref58] McMahon K. J. Castelli M. E. Vescovi E. G. Fieldman M. F. (2012). Biogenesis of outer membrane vesicles in *Serratia marcescens* is thermoregulated and can be induced by activation of the Rcs phosphorelay system. J. Bacteriol. 194, 3241–3249. doi: 10.1128/JB.00016-12 22493021 PMC3370869

[ref59] Morè N. Martorana A. M. Biboy J. Otten C. Winkle M. Serrano C. K. G.. (2019). Peptidoglycan remodeling enables *Escherichia coli* to survive severe outer membrane assembly defect. MBio 10, e02729–e02718. doi: 10.1128/mBio.02729-18 30723128 PMC6428754

[ref60] Moya-Beltrán A. Beard S. Rojas-Villalobos C. Issotta F. Gallardo Y. Ulloa R.. (2021). Genomic evolution of the class *Acidithiobacillia*: deep-branching Proteobacteria living in extreme acidic conditions. ISME J. 15, 3221–3238. doi: 10.1038/s41396-021-00995-x 34007059 PMC8528912

[ref61] Moya-Beltrán A. Rojas-Villalobos C. Díaz M. Guiliani N. Quatrini R. Castro M. (2019). Nucleotide second messenger-based signaling in extreme Acidophiles of the *Acidithiobacillus* species complex: partition between the Core and variable gene complements. Front. Microbiol. 10:381. doi: 10.3389/fmicb.2019.00381 30899248 PMC6416229

[ref9002] Ňancucheo I. Rowe O. F. Hedrich S. Johnson D. B. (2016). Solid and liquid media for isolating and cultivating acidophilic and acid-tolerant sulfate-reducing bacteria. FEMS Microbiol. Lett. 363:fnw083. doi: 10.1093/femsle/fnw083 27036143

[ref62] Nevot M. Deroncelé V. Messner P. Guinea J. Mercadé E. (2006). Characterization of outer membrane vesicles released by the psychrotolerant bacterium *Pseudoalteromonas antarctica* NF3. Environ. Microbiol. 8, 1523–1533. doi: 10.1111/j.1462-2920.2006.01043.x 16913913 PMC4379500

[ref63] Olofsson A. Vallström A. Petzold K. Tegtmeyer N. Schleucher J. Carlsson S.. (2010). Biochemical and functional characterization of *Helicobacter pylori* vesicles. Mol. Microbiol. 77, 1539–1555. doi: 10.1111/j.1365-2958.2010.07307.x 20659286 PMC3068288

[ref64] Partridge J. D. Harshey R. M. (2013a). More than motility: *Salmonella flagella* contribute to overriding friction and facilitating colony hydration during swarming. J. Bacteriol. 195, 919–929. doi: 10.1128/JB.02064-12 23264575 PMC3571333

[ref65] Partridge J. D. Harshey R. M. (2013b). Swarming: flexible roaming plans. J. Bacteriol. 195, 909–918. doi: 10.1128/JB.02063-12 23264580 PMC3571328

[ref66] Pérez-Cruz C. Carrión O. Delgado L. Martinez G. López-Iglesia C. Mercade E. (2013). New type of outer membrane vesicle produced by the gram-negative bacterium *Shewanella vesiculosa* M7T: implications for DNA content. Appl. Environ. Microbiol. 79, 1874–1881. doi: 10.1128/AEM.03657-12 23315742 PMC3592255

[ref67] Pérez-Cruz C. Delgado L. López-Iglesias C. Mercade E. (2015). Outer-inner membrane vesicles naturally secreted by gram-negative pathogenic bacteria. PLoS One 10:e0116896. doi: 10.1371/journal.pone.0116896 25581302 PMC4291224

[ref9003] Perez-Riverol Y. Bai J. Bandla C. Hewapathirana S. García-Seisdedos D. Kamatchinathan S. (2022). The PRIDE database resources in 2022: A Hub for mass spectrometry-based proteomics evidences. Nucleic. Acids. Res. 50:D543–D552. 34723319 10.1093/nar/gkab1038 PMC8728295

[ref68] Pierson T. Matrakas D. Taylor Y. U. Manyam G. Morozov V. Zhou W.. (2011). Proteomic characterization and functional analysis of outer membrane vesicles of *Francisella novicida* suggests possible role in virulence and use as a vaccine. J. Proteome Res. 10, 954–967. doi: 10.1021/pr1009756 21138299

[ref69] Pirbadian S. Barchinger S. E. Leung K. M. Byun H. S. Jangir Y. Bouhenni R. A.. (2014). *Shewanella oneidensis* MR-1 nanowires are outer membrane and periplasmic extensions of the extracellular electron transport components. Proc. Natl. Acad. Sci. U. S. A. 111, 12883–12888. doi: 10.1073/pnas.1410551111 25143589 PMC4156777

[ref70] Pollet H. Conrard L. Cloos A. S. Tyteca D. (2018). Plasma membrane lipid domains as platforms for vesicle biogenesis and shedding? Biomol. Ther. 8:94. doi: 10.3390/biom8030094 PMC6164003 30223513

[ref71] Remis J. P. Wei D. Gorur A. Zemla M. Haraga J. Allen S.. (2014). Bacterial social networks: structure and composition of *Myxococcus xanthus* outer membrane vesicle chains. Environ. Microbiol. 16, 598–610. doi: 10.1111/1462-2920.12187 23848955 PMC4234120

[ref72] Sand W. Gehrke T. (2006). Extracellular polymeric substances mediate bioleaching/biocorrosion via interfacial processes involving iron (III) ions and acidophilic bacteria. Res. Microbiol. 157, 49–56. doi: 10.1016/j.resmic.2005.07.012 16431087

[ref73] Sauer K. Stoodley P. Goeres D. M. Hall-Stoodley L. Burmølle M. Stewart P. S.. (2022). The biofilm life cycle: expanding the conceptual model of biofilm formation. Nat. Rev. Microbiol. 20, 608–620. doi: 10.1038/s41579-022-00767-0 35922483 PMC9841534

[ref74] Schooling S. R. Beveridge T. J. (2006). Membrane vesicles: an overlooked component of the matrices of biofilms. J. Bacteriol. 188, 5945–5957. doi: 10.1128/JB.00257-06 16885463 PMC1540058

[ref75] Schooling S. R. Hubley A. Beveridge T. J. (2009). Interactions of DNA with biofilm-derived membrane vesicles. J. Bacteriol. 191, 4097–4102. doi: 10.1128/JB.00717-08 19429627 PMC2698485

[ref76] Schwechheimer C. Kuehn M. J. (2015). Outer-membrane vesicles from gram-negative bacteria: biogenesis and functions. Nat. Rev. Microbiol. 13, 605–619. doi: 10.1038/nrmicro3525 26373371 PMC5308417

[ref77] Shinoda K. Tomita M. Ishihama Y. (2010). emPAI Calc--for the estimation of protein abundance from large-scale identification data by liquid chromatography-tandem mass spectrometry. Bioinformatics 26, 576–577. doi: 10.1093/bioinformatics/btp700 20031975

[ref78] Szczepaniak J. Holmes P. Rajasekar K. Kaminska R. Samsudin F. Inns P. G.. (2020). The lipoprotein pal stabilises the bacterial outer membrane during constriction by a mobilisation-and-capture mechanism. Nat. Commun. 11:1305. doi: 10.1038/s41467-020-15083-5 32161270 PMC7066135

[ref79] Tian C. M. Yang M. F. Xu H. M. Zhu H. M. Zhang Y. Yao J.. (2023). Emerging role of bacterial outer membrane vesicle in gastrointestinal tract. Gut Pathog. 15:20. doi: 10.1186/s13099-023-00543-2 37106359 PMC10133921

[ref80] Toyofuku M. Schild S. Kaparakis-Liaskos M. Eberl L. (2023). Composition and functions of bacterial membrane vesicles. Nat. Rev. Microbiol. 21, 415–430. doi: 10.1038/s41579-023-00875-5 36932221

[ref9004] Toyofuku M. Nomura N. Eberl L. (2019). Types and origins of bacterial membrane vesicles. Nat. Rev. Microbiol. 17, 13–24. doi: 10.1038/s41579-018-0112-2 30397270

[ref81] Tuffnell S. (2017). Acid drainage: the global environmental crisis you’ve never heard of. Available at: https://theconversation.com/acid-drainage-the-global-environmental-crisis-youve-never-heard-of-83515 (Accessed October 30, 2023).

[ref9005] Valdés J. Pedroso I. Quatrini R. Holmes D. S. (2008). Comparative genome analysis of Acidithiobacillus ferrooxidans, A. thiooxidans and A. caldus: Insights into their metabolism and ecophysiology. Hydrometallurgy, 94, 180–184. doi: 10.1016/j.hydromet.2008.05.039

[ref82] Vera M. Schippers A. Hedrich S. Sand W. (2022). Progress in bioleaching: fundamentals and mechanisms of microbial metal sulfide oxidation – part a. Appl. Microbiol. Biotechnol. 106, 6933–6952. doi: 10.1007/s00253-022-12168-7 36194263 PMC9592645

[ref83] Vera M. Schippers A. Sand W. (2013). Progress in bioleaching: fundamentals and mechanisms of bacterial metal sulfide oxidation-part A. Appl. Microbiol. Biotechnol. 97, 7529–7541. doi: 10.1007/s00253-013-4954-2 23720034

[ref84] Vriens B. Plante B. Seigneur N. Jamieson H. (2020). Mine waste rock: insights for sustainable hydrogeochemical management. Fortschr. Mineral. 10:728. doi: 10.3390/min10090728

[ref85] Wang W. Chanda W. Zhong M. (2015). The relationship between biofilm and outer membrane vesicles: a novel therapy overview. FEMS Microbiol. Lett. 362:fnv117. doi: 10.1093/femsle/fnv117 26208528

[ref86] Wei X. Vassallo C. N. Pathak D. T. Wall D. (2014). *Myxobacteria* produce outer membrane-enclosed tubes in unstructured environments. J. Bacteriol. 196, 1807–1814. doi: 10.1128/JB.00850-13 24391054 PMC4011004

[ref87] Weigand R. A. Vinci K. D. Rothfield L. I. (1976). Morphogenesis of the bacterial division septum: a new class of septation defective mutants. Proc. Natl. Acad. Sci. U. S. A. 73, 1882–1886. doi: 10.1073/pnas.73.6.1882 778849 PMC430411

[ref88] Yang J. Arratia P. E. Patteson A. E. Gopinath A. (2019). Quenching active swarms: effects of light exposure on collective motility in swarming *Serratia marcescens*. J. R. Soc. Interface 16:20180960. doi: 10.1098/rsif.2018.0960 31311436 PMC6685032

[ref89] Zhou X. Keller R. Volkmer R. Kraussm N. Scheerer P. Hunke S. (2011). Structural basis for two-component system inhibition and pilus sensing by the auxiliary CpxP protein. J. Biol. Chem. 286, 9805–9814. doi: 10.1074/jbc.M110.194092 21239493 PMC3059015

[ref90] Zhou L. Srisatjaluk R. Justus D. E. Doyle R. J. (1998). On the origin of membrane vesicles in gram-negative bacteria. FEMS Microbiol. Lett. 216, 217–222. doi: 10.1016/S0378-1097(98)00147-5 9673026

[ref91] Zielke R. A. Wierzbicki I. H. Weber J. V. Gafken P. R. Sikora A. E. (2014). Quantitative proteomics of the *Neisseria gonorrhoeae* cell envelope and membrane vesicles for the discovery of potential therapeutic targets. Mol. Cell. Proteomics 13, 1299–1317. doi: 10.1074/mcp.M113.029538 24607996 PMC4014286

