# Supplementary material for: Membrane vesicles in Acidithiobacillia class extreme acidophiles: influence on collective behaviors of ‘Fervidacidithiobacillus caldus’
Source: Front Microbiol. 2024 Jan 26;14:1331363. doi: 10.3389/fmicb.2023.1331363 (PMC10853474; doi:10.3389/fmicb.2023.1331363)
Supplement: Supplementary file 3 [file Data_Sheet_1.docx]

**Membrane Vesicles in Acidithiobacillia class extreme acidophiles: influence on collective behaviours of ‘*Fervidacidithiobacillus caldus*'. Rossoni et al.**

**Supplementary Methods**

**SM1. Swarming motility assay and PTSM medium**

*`F. caldus*´ ATCC 51756 and *A. thiooxidans* ATCC19377^T^ were grown in BART medium (pH 2.8) using 0.15% w/v tetrathionate (K_2_S_4_O_6_) as energy source for 5-7 days, when the cultures reached exponential (~8 x 10^7^ cells/ml) or stationary phase (~1.3 x 10^8^ cells/ml), respectively. Cells were washed twice with fresh BART medium, resuspended at 4 x 10^9^ cells/ml and inoculated in the center of swarming plates containing Phytagel-Tetrathionate Semisolid Medium (PTSM). PTSM for swarming assays was prepared by supplementing modified BART medium (pH 2.8) with tetrathionate (0.15 % w/v) as energy source and Phytagel (Sigma) (0.07 % w/v) as gelling agent. This media was suitable for surficial bacterial locomotion evaluation (yet hard enough to prevent cells immersion) according to extensive motility testing (Supplementary Figure S8). Plates were incubated at 30°C for *A. thiooxidans*^T^ and 40°C for `*F. caldus^´^*. Swarming was monitored daily using a stereoscope (LEICA EZ4 W) (10 X) and photographs were taken with a camera (Canon Power Shot G12) at a focal length of 21 cm.

**SM2. Differential staining setup of `*F. caldus*´ cells and MVs for epifluorescence microscopy (EFM)**

Differential staining was essential for semi-quantitative studies of cell and MVs on sulfur lentils. The low yield of DNA associated with isolated MVs isolated by our protocol (see point 2.6. in the main manuscript; 1.8 x 10^-9^ ng DNA/particle ~ 1.75 kbp/particle), prevented us from staining MVs with the DNA dye 4',6-diamidino-2-phenylindole (DAPI) in favour of *`F. caldus´* cell (Supplementary Figure S3). MVs were subjected to staining with diverse fluorescent dyes with protein (Sypro Orange) or lipid (FM4-64, DiA, DiI, DiO) binding affinities. Several of them yielded non-quantifiable results or poor signal (data not shown). Besides FM4-64 dye, which was highly efficient in staining both MVs and cells (Figure 1), we also obtained positive signals with DiOC18(3) (3,3'-Dioctadecyloxacarbocyanine Perchlorate) (DiO). After testing several concentrations (11.3, 22.6, or 55.6 µM), we determined that at 11.3 µM, DiO staining allowed the detection of MVs but showed no binding to *`F. caldus´* cells on sulfur (Supplementary Figure S4).

The post-attachment staining procedure with DiO did not significantly impact bacterial attachment or the detection of DNA using DAPI. Statistical analysis with Kruskal-Wallis' multiple comparisons test showed no significant differences in the density of recognized *`F. caldus´* cells, or their microcolonies on sulfur coupon surfaces in the presence or absence of DiO (adjusted *p*-value of 0.999). Images were acquired with an inverted EFM microscope Zeiss® Axio Observer Z1/7 equipped with objectives 20X (N.A. 0.35) and 40X (N.A 0.75). The microscope was equipped with the filters 38 HE Green fluorescent protein filter Ex BP 490/50 (HE), FT 495 (HE), Em BP 500/50 (HE), 43 HE DS Red Ex BP 562/38, FT 570, Em BP 640/570, 49 DAPI Ex BP 383/35, FT 395, Em BP 420/70, 71 Hc Red, Ex BP 580/604, FT 615, Em BP 625/725. Images were taken using the 40 X objective and the software Zeiss® Zen Pro version 3.0. For massive image analysis, at least 12 tiles/sample were taken, each composed of 16 individual images, taken in Z-stacking mode, with a focus depth of approximately 30 layers of 0.57 µm each.

**SM3. Quantification and Colocalization of *`F. caldus´* MVs and cells on sulfur surface**

Sulfur lentils were incubated with or without MVs at 2 concentrations (1X=7.5x10^9^ MVs and 10X=7.5x10^10^ MVs) and then exposed to a planktonic cell suspension (3-5 x 10^7^ cells/ml) for 120 min. After this, staining with DAPI (300 nM) and DiO (11.3 µM) was done. EFM images were manually pre-filtered and then processed using a Python-based code, which allowed for the extraction of quantitative parameters of interest (*particle sizes, channel, and quantity*). Several Python libraries were used, such as *os*, *pandas*, *matplotlib.pyplot*, *numpy, shutil, PIL, OpenCV, scipy.ndimage, matplotlib.figure, matplotlib* and *skimage* (*scikit-image*). Fluorescent channels were analysed, first loaded in grey scale. The background was subtracted by a rolling ball method (Van der Walt et al., 2014; Balatsko, 2018) and the image blurriness and contrast were evaluated (Van der Walt S. et al., 2014; Rosebrock, 2015). Next a binary image was formed from the fluorescent channels allowing us to identify and isolate large `blobs´ or other problem areas. If unsuitable for further processing due to contrast issues or due to the size of the fluorescent blob size, the images were filtered out. Problematic local areas of the images were registered and susbtrated from the total area analysed. To detect fluorescent blobs of interest, we used the *Laplacian of Gaussian* (LoG) from the *scikit-image* library (Van der Walt S. et al., 2014, Blob Detection), on grayscale-transformed fluorescent images. For further analyses, cell fluorescent signals were selected within an area range between 1 – 250 μm2, while MV signals were defined within an area range between 1 – 40 μm2. These values were selected by measuring single cells or MV’s signals in EFM images. Large clusters of cells or MVs were not accurately quantifiable, as it was not possible to single out each signal spot. Signals were considered as colocalized if the distance between the centres of two signal spots (DAPI for cells and DiO for MVs) was less than the sum of their radii. Colocalization between signal spots detected by DiO and DAPI channels showed that only 1.2-3.5% of these shared the same location on the sulfur surface, confirming that DiO (11.3 µM) dye stain MVs more effectively than cells. For images that included a secondary channel (brightfield), the same steps from image smoothing to thresholding were applied, this time to identify any imperfection on the sulfur surface. The data was analysed using GraphPad Prism software, employing SEM (Standard Error of the Mean) to evaluate differences between experimental conditions and establish correlations between variables (presence of vesicles, vesicle density, presence of *`F. caldus´* cells). Since MV´s tend to aggregate, signal spots were not efficiently recognized when MV’s particle density (7.5 x 10^10^ particles/ml) was used. Therefore, reliable data was obtained when adding a tenth of the MVs suspension (7.5 x 10^9^ particles/ml) (Supplementary Figure S9).

**SM4. Sample preparation for Mass Spectrometry**

MV samples (MV1 and MV2) were treated with protease/phosphatase inhibitor (Thermo Scientific, #1861284), lyophilized, and resuspended in 8 M urea with 25 mM of ammonium bicarbonate (pH 8.0). Next, the preparations were homogenized using ultrasound for 1 min in a cold bath, with 10 s pulses (on/off) at 50 % amplitude. Samples were incubated in ice for 5 min and centrifuged at 21,000 g for 10 min at 4°C to remove debris. Samples were quantified immediately using the Qubit Protein Assay Reagent (Invitrogen, #Q33212). Total proteins (20 μg) were resuspended in 40 μL of loading buffer and denatured for 10 min at 95°C. Then, samples were subjected to electrophoresis under denaturing conditions (SDS-PAGE) in a 10 % polyacrylamide gel, at 100 V. The resultant gel was stained overnight with 50 mL of biocompatible Coomasie R-250 (Bio Rad, (#161-0786) at room temperature (Supplementary Figure S5). Proteins were subjected to chloroform methanol extraction, dried on a rotary concentrator and resuspended in 30 μL 8 M Urea and 25 mM ammonium bicarbonate. Proteins were reduced with DTT to a final concentration of 20 mM in 25 mM ammonium bicarbonate and incubated for 1 h at room temperature. Samples were alkylated by adding iodoacetamide to a final concentration of 20 mM in 25 mM ammonium bicarbonate, and incubated for 1 h in darkness at room temperature. Subsequently, the samples were diluted 8 times with 25 mM ammonium bicarbonate. Digestion was performed with sequencing grade Trypsin (Promega, #V5071) in a 1:50 ratio of protease:protein (mass / mass) during 16 h at 37°C. The reaction was stopped by acidification, adding 10 % formic acid. Then, the samples were submitted to a Clean-up Sep-Pack C18 Spin Columns (Waters Milford) according with the supplier recommendations (for reversed-phase sorbents, preconditioning of the sorbent with an organic solvent i.e., methanol, acetonitrile, isopropanol, or tetrahy- drofuran was done). Subsequently, the clean peptides were dried in a rotary concentrator (hypervac vc2200 coupled to hyoercool hc3110) at 1,000 rpm overnight at 10°C.

**SM5. LC-MS/MS (Liquid Chromatography – Tandem Mass Spectometry)**

The peptides obtained (200 ng) were injected into a nanoUHPLC nanoElute (Bruker Daltonics) coupled to a timsTOF Pro mass spectrometer (`Trapped Ion Mobility Spectometry – Quadrupole Time Of Flight Mass Spectrometer´, Bruker Daltonics) using an Aurora UHPLC column (25 cm x 75 μm ID, 1.6 μm C18, IonOpticks, Australia). The liquid chromatography was performed using a 90-min gradient of 2 % to 35 % Buffer B (0.1 % Formic acid – Acetonitrile). The collection of results was carried out using TimaControl 2.0 software (Bruker Daltonics) under 10 PASEF cycles, with a mass range of 100-1700 m/z ionization of the capillary 1,500 V and a temperature of 180°C in the capillary, TOF frequency 10 KHz at 50,000 FWHM resolution. Data obtained was analysed with the PEAKS Studio X + software (Bioinformatics Solutions). Mass tolerance parameters of 50 ppm were used, using monoisotopic masses, and Ionic fragments of 0.05 Da. Trypsin was used as enzyme for specific digestion mode, with a maximum of 2 cleavage losses per peptide (`missed cleavage per peptides´).

**Membrane Vesicles in Acidithiobacillia class extreme acidophiles: influence on collective behaviours of ‘*Fervidacidithiobacillus caldus*'. Rossoni et al.**

**Supplementary Figures**

**Figure S1.** MVs production of `*F. caldus*´ at different temperatures. `*F. caldus*´ cells (5x10^11^) were incubated for 8 h at physiological (40ºC) and stress conditions temperatures (55ºC and 70ºC). The experiment was performed in duplicates.

**Figure S2.** Early attachment of `*F. caldus´* on sulfur lentil surfaces. The time for early attachment was determined comparing the remaining planktonic cells exposed (red dots) and unexposed (black dots) to sulfur lentils. The early attachment effect is observed until 120 min.

**Figure S3.** EFM for determining MVs staining. MVs (7.5 x 10^10^ particles) were incubated for 2 h on sulfur lentils. Samples were stained with DAPI (300 mM) and DiO (11.3 μM) as described. Representative images, taken on tiles are shown. A) DAPI channel, B) Surface light reflection channel, C) DiO channel, D) Merged image of channels A-C. Size bars represent 100 μm


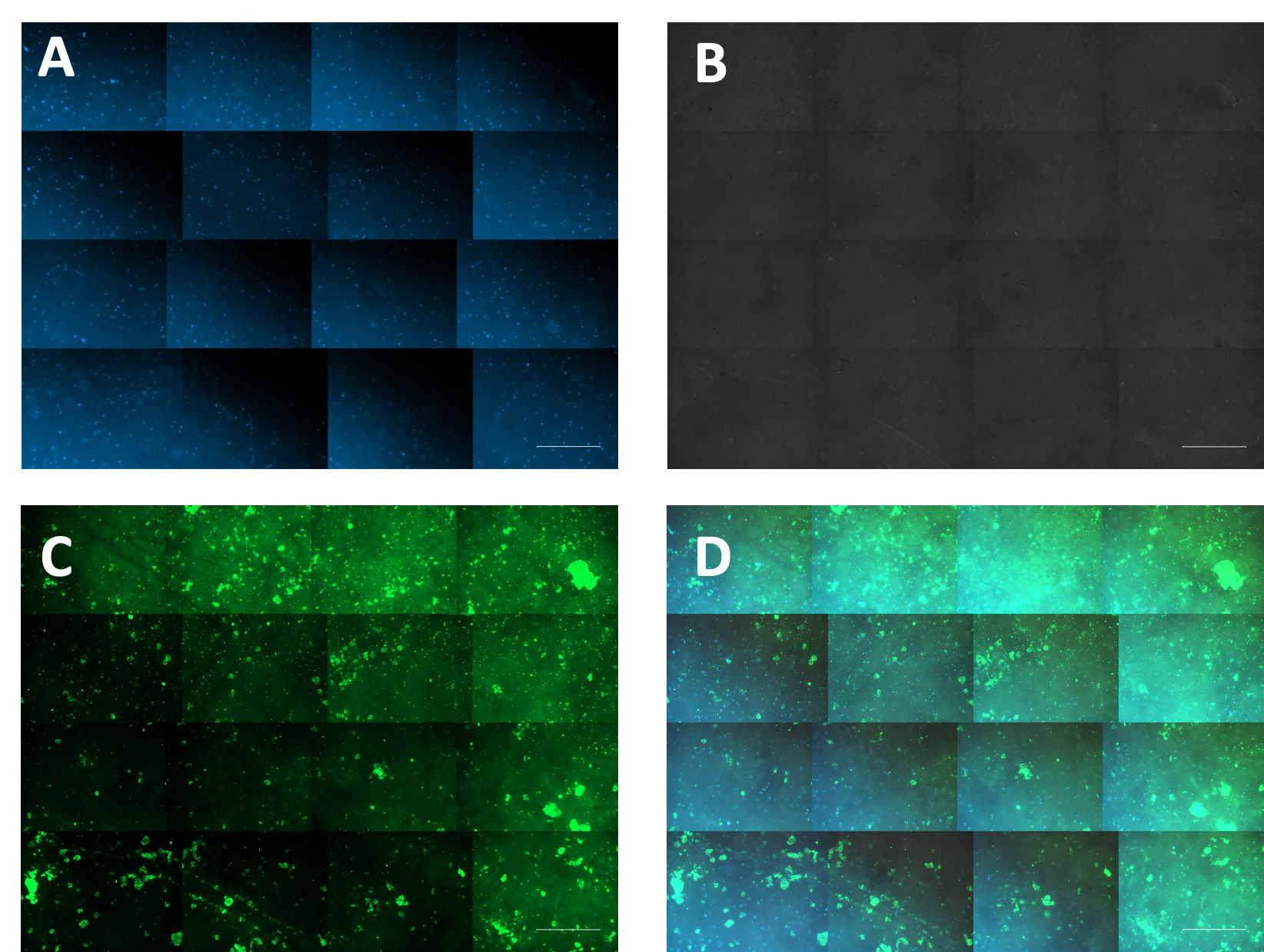


**Figure S4.** Differential fluorescent staining between `*F. caldus´* and MVs. MVs (7.5 x 10^10^ particles) were incubated for 2 h on sulfur lentils and afterwards 5 x 10^7^ cells/mL were added and incubated for 100 min. Samples were double-stained with DAPI (300 mM) and DiO (11.3 μM) as described. Representative images, taken on tiles are shown. A) DAPI channel, B) Surface light reflection channel, C) DiO channel, D) Merged image of channels A-C. Size bars represent 100 μm.


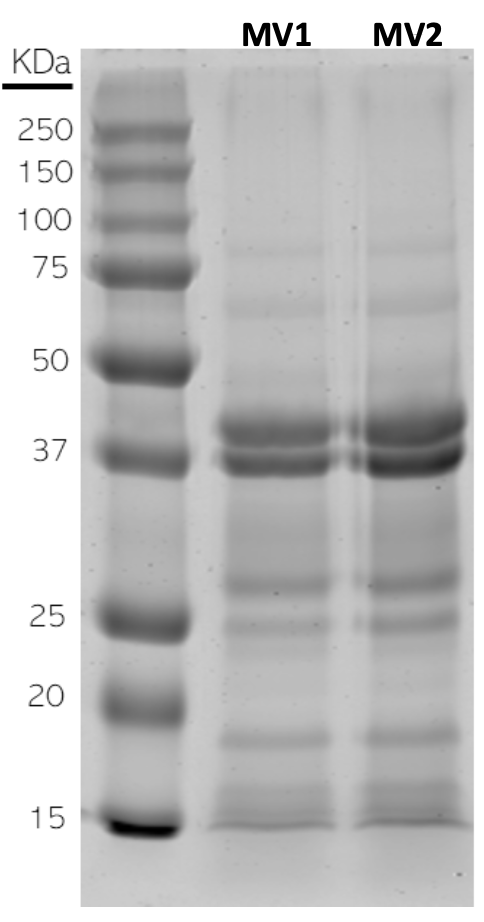


**Figure S5.** SDS-page showing proteins extracted from two biological replicates of `*F. caldus´* MVs purification (MV1 and MV2). 12.5% acrylamide gel were load with 20 mg of protein.

**Figure S6.** Identification of MVs-carried DNA. A) Membrane vesicles co-staining with DAPI (800 nM) for DNA and FM 4-64 (5 μg/mL) for membranes reveal DNA content. B) DNAse I resistant DNA extraction from `*F. caldus´* MVs. C) PCR amplification of markers for different compartment of `*F. caldus´* genome using specific primers for chromosome and mobile genetic elements.

**Figure S7.** Relative quantification and COG classification of `*F. caldus´* MV proteins samples. A) Sample MV1 (407 proteins), B) Sample MV2 (426 proteins). For each sample, the diversity and the protein content are depicted in left and right intra-panel, respectively.

**Figure S8.** Swarming motility in *Acidithiobacillaceae* family members. A) Swarming patterns on PTSM. `*F. caldus´*, and *A. thiooxidans*^T^ obtained from middle exponential phase cultures were inoculated on the surface of PTSM, showing swarming motility patterns in these two species. B) TEM observation of cells located in the migrating edge showed a single polar flagellum, indicating that the swarming motility of these three *Acidithiobacillaceae* family members is propelled by this organelle. It was also possible to distinguish MVs (red arrow) in swarming cell surroundings, suggesting these nanoparticles are important components for flagellar-based swarming motility.  Besides MVs, it is possible to observe electrodense material covering the cells, probably composed by extracellular polymeric substances (EPS) (blue arrow). Black bar, 1 mm.

**Figure S9.**Comparative MV staining with DiO, influence of the addition of `*F. caldus´* and the amount of MVs added. `*F. caldus`* was added to elemental sulfur lentils, represented by the green bars (with DiO staining) and grey bar (without DiO staining). MVs amount added were 7.5 x 10^10^ (10X, orange border) and 7.5 x 10^9^ particles/mL (1X, yellow border), respectively. The white bar represents a MVs and cell-free control (white bar). All signals were recorded only in the DiO channel. The quantification was accomplished via particle/microcolony counting per unit of area (μm2). A total of 951 images were quantified between these conditions.

**Membrane Vesicles in Acidithiobacillia class extreme acidophiles: influence on collective behaviours of ‘*Fervidacidithiobacillus caldus*'. Rossoni et al.**

**Supplementary References**

Balatsko, M. (2018). Rolling ball and sliding paraboloid background subtraction algorithms. PyPI. https://pypi.org/project/opencv-rolling-ball/

Rosebrock, A. (2015). Blur detection with OpenCV. Pyimagesearch. https://pyimagesearch.com/2015/09/07/blur-detection-with-opencv/

van Der Walt, S., Schönberger, J.L., Nunez-Iglesias, J., Boulogne, F., Warner, J.D., Yager, N. *et al.* (2014). Scikit-image: Image processing in python. *PeerJ*. 2:e453. doi: 10.7717/peerj.453.
